# Recent Advances on Transition‐Metal‐Based Layered Double Hydroxides Nanosheets for Electrocatalytic Energy Conversion

**DOI:** 10.1002/advs.202207519

**Published:** 2023-03-03

**Authors:** Yuchen Wang, Man Zhang, Yaoyu Liu, Zhikeng Zheng, Biying Liu, Meng Chen, Guoqing Guan, Kai Yan

**Affiliations:** ^1^ Guangdong Provincial Key Laboratory of Environmental Pollution Control and Remediation Technology School of Environmental Science and Engineering Sun Yat‐sen University Guangzhou 510275 China; ^2^ Energy Conversion Engineering Laboratory Institute of Regional Innovation (IRI) Hirosaki University 3‐Bunkyocho Hirosaki 036‐8561 Japan

**Keywords:** electrocatalysts, energy conversion, nanosheets, reaction mechanism, transition‐metal‐based layered double hydroxides

## Abstract

Transition‐metal‐based layered double hydroxides (TM‐LDHs) nanosheets are promising electrocatalysts in the renewable electrochemical energy conversion system, which are regarded as alternatives to noble metal‐based materials. In this review, recent advances on effective and facile strategies to rationally design TM‐LDHs nanosheets as electrocatalysts, such as increasing the number of active sties, improving the utilization of active sites (atomic‐scale catalysts), modulating the electron configurations, and controlling the lattice facets, are summarized and compared. Then, the utilization of these fabricated TM‐LDHs nanosheets for oxygen evolution reaction, hydrogen evolution reaction, urea oxidation reaction, nitrogen reduction reaction, small molecule oxidations, and biomass derivatives upgrading is articulated through systematically discussing the corresponding fundamental design principles and reaction mechanism. Finally, the existing challenges in increasing the density of catalytically active sites and future prospects of TM‐LDHs nanosheets‐based electrocatalysts in each application are also commented.

## Graphical abstract

Current fabrication strategies to design transition‐metal‐based layered double hydroxides (TM‐LDHs) nanosheets are summarized. The electrocatalytic applications of these as‐fabricated TM‐LDHs nanosheets in oxygen evolution reaction, hydrogen evolution reaction, urea oxidation reaction, nitrogen reduction reaction, small molecule oxidation, and biomass derivatives upgrading are articulated through systematically discussing the corresponding fundamental design principles and reaction mechanism.

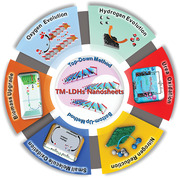

## 1 Introduction

**Figure 1:**
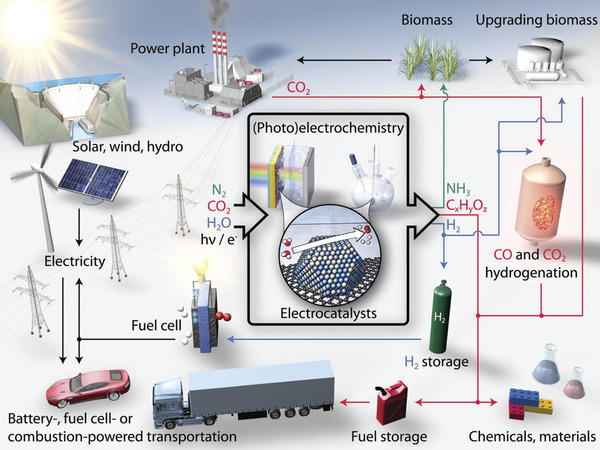
Schematic of a renewable energy landscape related to electrocatalysis. Reproduced with permission.^[^ ^3a^ ^]^ Copyright 2017, American Association for the Advancement of Science.

An increasing demand of energy and high‐value‐added chemicals for the growth and development of modern society has inevitably accelerated the consumption of limited nonrenewable fossil resources.^[^ ^1^ ^]^ The excessive carbon emission from fossil fuels has caused adverse changes to the climate, especially global warming issue.^[^ ^2^ ^]^ At this moment, renewable energy‐based technologies are urgently needed to achieve a sustainable development of human society (**Figure** **1**).^[^ ^3^ ^]^ Among manifold frontal technologies, those renewable energy powered electrocatalytic conversion techniques including water electrolysis and biomass upgrading have drawn tremendous interests owing to their high efficiency and nonpollution.^[^ ^4^ ^]^ Accordingly, the architecture of high‐performance electrocatalyst is essential to the efficient utilization of green energy and production of valuable chemicals.

**Figure 2:**
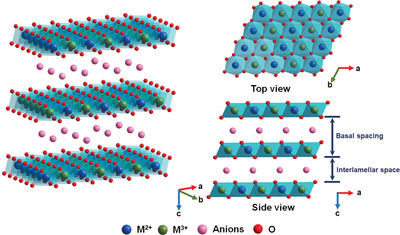
Schematic crystalline structure of TM‐LDHs.

2D materials are considered as desired alternatives to noble metal‐based electrocatalysts because of their unique electronic and anisotropic properties.^[^ ^5^ ^]^ Transition‐metal‐based layered double hydroxides (TM‐LDHs) are classical 2D electrocatalysts with earth abundance, cheapness, low toxicity, and high intrinsic electrocatalytic activity.^[^ ^6^ ^]^ As displayed in **Figure** **2**, TM‐LDHs possess a lamellar structure with the charge balance between transition metal divalent/trivalent cations (M^2+^/M^3+^) in host layers and anions (A*^n^* ^−^) in interlayers.^[^ ^7^ ^]^ A versatile formula of TM‐LDHs is expressed as [M^2+^_1−_ *_x_* M^3+^ *_x_* (OH)_2_]*^x^* ^+^[(A*^n^* ^−^)*_x_*_/_ *_n_* ·*y*H_2_O]*^x^* ^−^, where *x* can be valued in the range of 0.20–0.33.^[^ ^8^ ^]^ As such, the adjustment of interlayer spacing and the modulation of electronic structure can be realized by tuning transition metal divalent/trivalent cations and anions in TM‐LDHs.

Although TM‐LDHs have been intensively studied in the electrocatalysis field, insufficient active sites and limited electronic/ionic transport are main obstacles for their industrial applications.^[^ ^9^ ^]^ To effectively overcome these shortcomings, realizing TM‐LDHs nanosheets with nanometer‐level thickness and reducing lateral size are necessary.^[^ ^10^ ^]^ At the same time, it is reported that abundant defects are always generated in the prepared TM‐LDHs nanosheets, which are imperative for improving the intrinsic activity.^[^ ^11^ ^]^ Moreover, considering the lack of interlayer anions, TM‐LDHs nanosheets with positively charge are prone to hybrid with negatively charged nanostructured materials (e.g., graphene, carbon nanotubes, metal oxides).^[^ ^12^ ^]^ In addition, the electrostatic strengthened hybrid structure is beneficial for alleviating the agglomeration of TM‐LDHs nanosheets, enhancing the overall conductivity and ameliorating the electronic structure, giving rise to the optimized electrocatalytic performance.^[^ ^13^ ^]^

**Figure 3:**
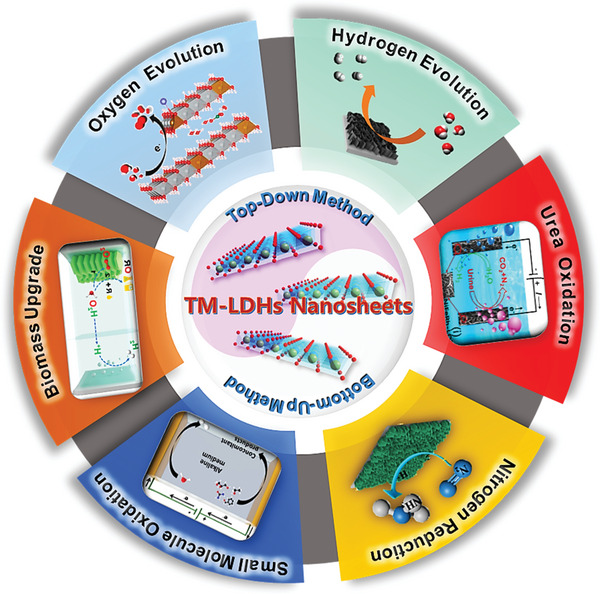
Schematic representation of synthetic strategies and electrocatalytic applications of TM‐LDHs nanosheets.

In this review, a brief overview of recent advances in the development and utilization of TM‐LDHs nanosheets for electrocatalytic energy conversion is presented (**Figure** **3**). It is proposed that TM‐LDHs nanosheets could be a promising category of energy materials in various electrocatalytic reactions including water splitting, urea oxidation, nitrogen reduction reaction, small molecule oxidation, and biomass derivatives upgrading. Herein, state‐of‐the‐art fabrication methodologies of TM‐LDHs nanosheets are first given and followed by a summary of the latest theoretical studies and experimental progress of TM‐LDHs nanosheets for electrocatalytic energy conversion. Especially, the applications of TM‐LDHs nanosheets in water splitting, biomass monomers upgrading are focused. In the end, the challenges and corresponding future perspectives of TM‐LDHs nanosheets as high‐performance electrocatalysts are proposed.

## 2 Synthetic Strategies for TM‐LDHs Nanosheets

In recent years, enormous synthetic strategies have been developed for fabricating TM‐LDHs nanosheets, which are generally classified into top‐down and bottom‐up strategies.^[^ ^14^ ^]^ The top‐down strategy is typically referred as exfoliation or delamination of conventional bulk TM‐LDHs to produce nanosheet structure using physical and chemical means. On the contrary, the bottom‐up strategy is a direct preparation route of TM‐LDHs nanosheets without presynthesizing bulk precursors. In this section, all relevant progresses on these two strategies are reviewed.

### 2.1 Top‐Down Strategy

Up to date, the top‐down strategy has been mostly used to achieve TM‐LDHs nanosheets by breaking the interlayer bonds in bulk parent crystal. In 1999, Adachi‐Pagano et al. achieved the first successful exfoliation of TM‐LDHs, in which the parent ZnAl‐LDHs were initially modified via the intercalation of dodecyl sulfate surfactants and then dispersed in butanol for 16 h.^[^ ^15^ ^]^ As such, the exfoliated ZnAl‐LDHs nanosheets could maintain stable for more than 8 months. It is also reported that other solvents (e.g., water, methanol, ethanol, propanol, or hexane) only partially delaminated ZnAl‐LDHs, revealing the importance of solvent type on the stripping effect.

**Figure 4:**
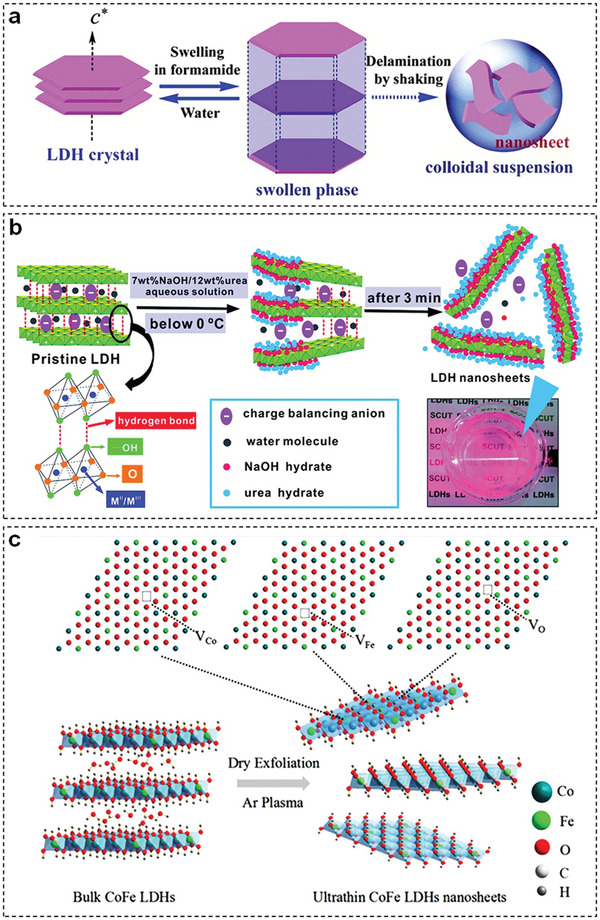
a) Exfoliation mechanism of TM‐LDHs in formamide. Reproduced with permission.^[^ ^17^ ^]^ Copyright 2006, American Chemical Society. b) Exfoliation of TM‐LDHs in sodium hydroxide/urea aqueous solution. Reproduced with permission.^[^ ^19^ ^]^ Copyright 2014, Royal Society of Chemistry. c) Schematic diagram of Ar plasma exfoliation of CoFe‐LDHs. Reproduced with permission.^[^ ^21^ ^]^ Copyright 2017, Wiley.

Among various solvents, formamide has been considered as a general exfoliation agent for TM‐LDHs.^[^ ^16^ ^]^ Liu et al. proposed a universal exfoliation mechanism of TM‐LDHs in formamide based on experimental results (**Figure** **4**a), in which TM‐LDHs first undergo a quick swelling process to weaken the strong hydrogen bonds between carbonyl groups of formamide and hydroxyl groups of TM‐LDHs slabs, and as such, the subsequent exfoliation process can proceed under continuous mechanical treatments to obtain TM‐LDHs nanosheets.^[^ ^17^ ^]^ Inspired by this, myriad mono‐ or few‐layer TM‐LDHs (e.g., NiCo‐, NiFe‐, NiV‐, NiAl‐, CoFe‐LDHs) nanosheets and their heterostructures have been successfully obtained on the basis of a delamination approach.^[^ ^10^, ^18^ ^]^

Apart from using poisonous polar organic solvents, water is also utilized as a mild solvent for liquid exfoliation. For example, Wei et al. introduced sodium hydroxide/urea aqueous solution to derive ZnAl‐LDHs nanosheets with a thickness of 0.6 nm at −10 °C.^[^ ^19^ ^]^ As seen in Figure 4b, the insertion of sodium hydroxide/urea hydrates can destroy the original hydrogen bond network of Zn‐Al LDHs and then promote the rapid delamination process at a subzero temperature. Lately, Chen et al. applied a novel hydrothermal approach to achieve NiFe‐LDHs nanosheets based on the Ostwald ripening phenomenon.^[^ ^20^ ^]^ Herein, compared with other liquid exfoliation strategies, bulk NiFe‐LDHs with different sizes are first grown on the copper mesh and then delaminated via a dissolve–redeposition process without using any surfactant and organic solvent. After an optimal hydrothermal period of 8 h, NiFe‐LDHs nanosheets with a thickness of 7−9 nm and a lateral size of 1 µm can be ultimately collected. Although above methods have successfully used to synthesize TM‐LDHs nanosheets, conventional liquid exfoliation techniques are still suffered from tediously long processing time and reaggregation of exfoliated nanosheets. Therefore, high‐efficient and eco‐friendly exfoliating strategies for the fabrication of stable ultrathin TM‐LDHs nanosheets are urgently needed.

Plasma technology has been widely used to exfoliate layered materials such as graphite,^[^ ^22^ ^]^ transition metal oxides,^[^ ^23^ ^]^ and also TM‐LDHs.^[^ ^21^ ^]^ For a typical plasma exfoliation of TM‐LDHs, the bulk TM‐LDHs can be initially synthesized through a hydrothermal method and then attacked by plasma‐induced active particles under a certain atmosphere (e.g., Ar, N_2_, O_2_). Herein, these active particles will break the chemical interaction between host layers so that corresponding interlayer anions can separate TM‐LDHs lamellar structure. Such a plasma exfoliation technique has the advantages of cleanness, time‐saving, and low energy consumption in comparison with the traditional liquid exfoliation technique.^[^ ^24^ ^]^ In 2017, Wang et al. first applied Ar plasma to obtain ultrathin CoFe‐LDHs nanosheets (Figure 4c).^[^ ^21^ ^]^ It is found that the plasma etching process not only reduced the thickness of CoFe‐LDHs from 20 to 0.6 nm but also generated Co, Fe, and O vacancies, which are advantageous to the electrocatalytic performance. Later on, Liu et al. successfully delaminated NiCo‐LDHs via 15 s of Ar plasma treatment.^[^ ^25^ ^]^ To expand the application of this technique, N_2_ and water plasma have been also utilized to realize the fabrication of CoFe‐LDHs and CoAl‐LDHs nanosheets.^[^ ^26^ ^]^

### 2.2 Bottom‐Up Strategy

Compared with the top‐down strategy, the bottom‐up strategy is relatively simple to directly synthesize TM‐LDHs nanosheets from transition metal salts. To date, various bottom‐up methods including reverse microemulsion method, co‐precipitation method with nucleation/growth controlled inhibitor, pulsed‐laser ablation, and ultrasonic/mechanical stirring methods have been developed.

**Figure 5:**
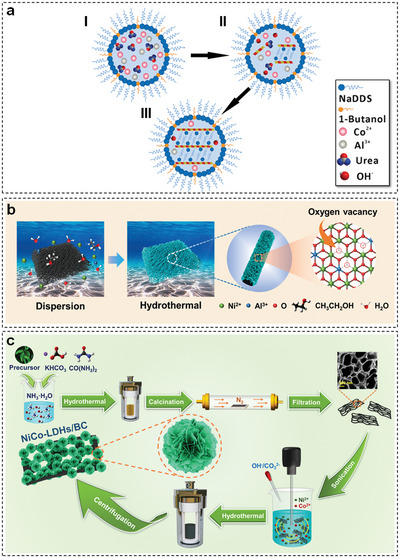
a) Schematic representation of the formation of TM‐LDHs nanosheets in microemulsion. Reproduced with permission.^[^ ^27^ ^]^ Copyright 2011, American Chemical Society. b) One‐step hydrothermal strategy used for fabricating oxygen‐deficient NiAl‐LDH nanosheets. Reproduced with permission.^[^ ^30^ ^]^ Copyright 2022, Science China Press. c) In situ growth of NiCo‐LDHs nanosheets on porous carbon materials. Reproduced with permission.^[^ ^39^ ^]^ Copyright 2022, KeAi Publishing Communications Ltd.

The reverse microemulsion approach is effective for preparing TM‐LDHs nanosheets via the introduction of aqueous co‐precipitation system into oil phase. Generally, the fabrication process is illustrated in **Figure** **5**a: 1) transition metal ions and urea are mixed in micelles, which are surrounded by surfactant; 2) TM‐LDHs are generated along with the slow hydrolysis of urea; 3) the formation of TM‐LDHs nanosheets in a confined environment.^[^ ^27^ ^]^ Herein, the water/surfactant molar ratio can be adjusted to control the size of TM‐LDHs nanosheets. For instance, Zhao et al. applied the reverse microemulsion method to obtain NiTi‐LDH nanosheets, which possessed a thickness of 0.9 nm with a particle size of 20 nm.^[^ ^28^ ^]^ However, it should be noted that long reaction time and difficult removal of surfactants cannot be ignored by using this approach.

As regards the traditional co‐precipitation or hydrothermal process, numerous inhibitors including formamide, ethyl alcohol, and ethylene glycol are added to weaken the interactions between TM‐LDHs host layers, thereby preventing their growth along the *c*‐axis. For example, Zhao et al. applied a co‐precipitation method to obtain monolayer NiFe‐LDHs precursors with a thickness of 1.0 nm and a lateral size of 30 nm in formamide.^[^ ^29^ ^]^ The subsequent ultrasonication effectively reduced the corresponding sizes to 0.6 and 1.5−3.0 nm, respectively. Wang et al. adopted a facile hydrothermal method with the aid of ethyl alcohol to construct oxygen‐deficient NiAl‐LDHs nanosheets on carbon fiber paper (Figure 5b), in which the growth of NiAl‐LDHs nanosheets proceeded without adding any alkaline source.^[^ ^30^ ^]^ The obtained petal‐like NiAl‐LDHs nanosheets provided a high specific surface area with desired intrinsic conductivity, which is vital to achieve superior electrocatalytic performance. Li et al. successfully prepared stable single‐layer CoAl‐LDHs using a hydrothermal method in ethylene glycol.^[^ ^31^ ^]^ It is considered that the formation of the single‐layer CoAl‐LDHs should be mainly attributed to the inherent chelation and absorption ability of ethylene glycol.

To avoid the use of toxic organic solvents, the electrodeposition method and mechanical strategies including laser ablation and ultrasonic/mechanical stirring methods have been extensively reported to prepare TM‐LDHs nanosheets. Electrodeposition method is a facile and eco‐friendly process to fabricate TM‐LDHs nanosheets on suitable conductive substrates. Typically, nitrate ions in the electrolyte are reduced under constant current or potential to produce hydroxide ions, which can further combine with transition metal cations to form TM‐LDHs nanosheet arrays on the surface of the substrate.^[^ ^6^, ^32^ ^]^ The morphologies of as‐prepared TM‐LDHs nanosheets are highly dependent on electrodeposition parameters, such as reaction time and precursor concentration.^[^ ^33^ ^]^ Wang et al. employed in situ liquid‐phase transmission electron microscopy to investigate the influence of electrodeposition parameters on the growth mode and density of CoFe‐LDHs nanosheets.^[^ ^34^ ^]^ It is found that the growth of CoFe‐LDHs nanosheets was rapid at the initial stage and then unchanged after reaching a critical areal coverage. Impressively, the vertical or lateral growth of these nanosheets could be modulated by the precursor concentration, providing a guidance for controllable electrochemical synthesis of TM‐LDHs nanosheets.

With respect to mechanical strategies, Hunter et al. employed the pulse‐laser ablation technique to prepare NiFe‐LDHs nanosheets as follows: 1) NiFe‐LDHs precursors and pure metallic powders are magnetically stirred in alkaline water (pH = 10); 2) the mixed solution is irradiated to plasma state by a pulse‐laser beam for 60 min; 3) NiFe‐LDHs nanosheets are formed by supercooling and subsequent condensation of the plasma.^[^ ^35^ ^]^ Gao et al. expanded the application of laser ablation technique to synthesize CoO/CoFe‐LDHs nanocomposites in sodium chloride solution.^[^ ^36^ ^]^ Herein, during the irritation process, Co‐Fe alloy target was activated and reacted with chloride and hydroxide ions to generate CoFe‐LDHs nanosheets, and at the same time, excessive Co^2+^ could interact with water to form CoO on the surface of CoFe‐LDHs. It is believed that the CoFe‐LDHs content in composites was determined by the concentration of sodium chloride. Except for the laser ablation method, Chen et al. first proposed a ultrasonication/mechanical stirring method to obtain NiCr‐LDHs nanosheets without adding any growth inhibitor, in which the ultrasonication/mechanical stirring treatment acted as a mechanical inhibitor to greatly reduce the thickness of NiCr‐LDHs during the co‐precipitation process.^[^ ^37^ ^]^ Afterward, Wang et al. used the same method to successfully synthesize NiFe‐LDHs nanosheets and studied the influence of ultrasonic time on their thickness.^[^ ^38^ ^]^ With increasing ultrasonic time, the thickness of NiFe‐LDHs decreased from 61.3 to 7.4 nm. However, excessive ultrasonic treatment partially destroyed the nanosheet structure of NiFe‐LDHs, leading to fragmental morphology and increased thickness. Furthermore, Wang et al. applied a similar methodology to controllably grow NiCo‐LDHs nanosheets on the surface of porous carbon, which were derived from *Dicranopteris dichotoma* stems.^[^ ^39^ ^]^ As seen in Figure 5c, porous carbon and Ni^2+^/Co^2+^ ions were mixed in a beaker and the titration process was carried out under the ultrasonication/mechanical stirring. The mechanical treatment could effectively prevent the aggregation of NiCo‐LDHs nanosheets to realize their rational distribution on porous carbon. Considering the simplicity and high efficiency, the above‐mentioned mechanical strategies are promising for large‐scale preparation of TM‐LDHs nanosheets.

In the following, the utilization of these fabricated TM‐LDHs nanosheets for oxygen evolution reaction (OER), hydrogen evolution reaction (HER), urea oxidation reaction (UOR), nitrogen reduction reaction (NRR), small molecule oxidation, and biomass derivatives upgrading are articulated through systematically discussing the corresponding fundamental design principles and reaction mechanism.

## 3 Electrocatalytic Water Splitting

### 3.1 Oxygen Evolution Reaction

#### 3.1.1 Mechanism

In state‐of‐art electrochemical energy systems (e.g., fuel cell, water electrolyzer, metal–air battery), OER is the key reaction to determine their overall performance.^[^ ^40^ ^]^ Commonly, OER with sluggish four‐electron transfer proceeds through absorption/desorption of different oxygen intermediates. Deciphering the mechanism of OER is beneficial to develop efficient and robust OER electrocatalysts.

**Figure 6:**
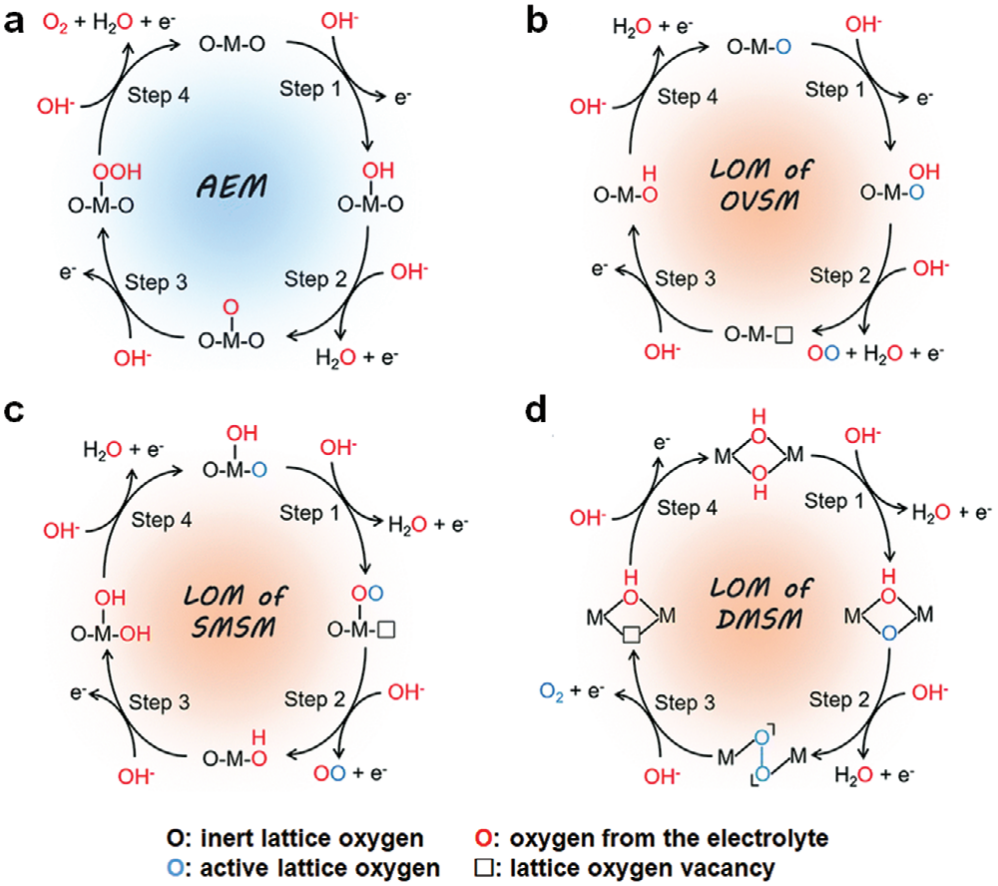
The schematic illustration of AEM and LOM pathways for alkaline OER. Reproduced with permission.^[^ ^44^ ^]^ Copyright 2021, Royal Society of Chemistry.

At present, two mainstream pathways of OER have been proposed as adsorbate evolution mechanism (AEM) and lattice oxygen mediated mechanism (LOM). **Figure** **6** distinctly illustrates these pathways. For the AEM pathway (Figure 6a), OH^−^ undergoes adsorption/deprotonation/coupling/desorption steps to release O_2_ with the generation of OH*, O*, and OOH* intermediates.^[^ ^41^ ^]^ These four steps are all thermodynamically unfavorable, resulting in a high overpotential to drive OER.^[^ ^42^ ^]^ Based on theoretical calculation and experimental results, the binding energies of OH*, O*, and OOH* intermediates are found to be linearly correlated on the basis of the following relationship, Δ*G*_OOH*_ = Δ*G*_OH*_ + 3.2 ± 0.2 eV.^[^ ^43^ ^]^ Considering the overall energy barrier of deprotonation/coupling step (2.46 eV), the overpotential limit of OER is determined as (3.2−2.46)/2 = 0.37 V. Nevertheless, the overpotential of substantial reported OER electrocatalysts is lower than the limited value of AEM pathway. Under this circumstance, an LOM pathway to trigger activated lattice oxygen in OER electrocatalysts is proposed, and further classified into oxygen‐vacancy, single‐metal, and dual‐metal site mechanisms (OVSM, SMSM, and DMSM).^[^ ^44^ ^]^ For the OVSM pathway (Figure 6b), OOH* intermediates are initially formed on single metal active sites by combining activated lattice oxygen and OH^−^.^[^ ^45^ ^]^ After releasing oxygen molecules, the resulting oxygen vacancies are refilled by OH^−^ to regenerate these active sites. Alternatively, the single metal active site in the SMSM pathway (Figure 6c) first absorbs OH^−^ to generate OH* and O* intermediates following the first and second steps of AEM, respectively. Afterward, O* intermediates are coupled with the activated lattice oxygen to form OO* intermediates, which are subsequently oxidized to release O_2_.^[^ ^46^ ^]^ Apart from single active site, O_2_ in the DMSM pathway (Figure 6d) are generated from OO* intermediates, which are formed by direct coupling of adjacent lattice oxygen from two metal active sites.^[^ ^47^ ^]^ Albeit LOM pathway involving O–O coupling bypass the theoretical limit of AEM pathway, the deprotonation energy is inevitably increased with the switch from AEM pathway to LOM pathway.^[^ ^48^ ^]^ For this reason, new pathways with advantages of both AEM and LOM should be further explored.

#### 3.1.2 Research

TM‐LDHs nanosheets have been extensively investigated as efficient electrocatalysts for OER. At the early stage, pristine TM‐LDHs nanosheets present outperformed OER activity in comparison with those commercial noble metal electrocatalysts. For example, Song et al. exfoliated a series of TM‐LDHs (NiCo‐, NiFe‐, and CoCo‐LDHs) in formamide to achieve nanosheets, which exhibited excellent OER activity.^[^ ^10b^ ^]^ In comparison with bulk TM‐LDHs, the enhanced electrocatalytic activity of nanosheets was mainly originated from the exposure of more active sites and the higher electronic. As such, NiFe‐LDHs nanosheets achieved the best OER activity with an overpotential of 302 mV, which was 36 mV lower than that of IrO_2_. The role of transition metal for OER was then unveiled by systematically studying four different transition metal (oxy)hydroxides.^[^ ^49^ ^]^ Experimental results demonstrated that OER activities of transitional metal elements were negatively correlated to their OH‐M^2+^ *^*δ*^* energetic strength. The trend established the fundamental criteria for designing active center of TM‐LDHs nanosheets.

**Figure 7:**
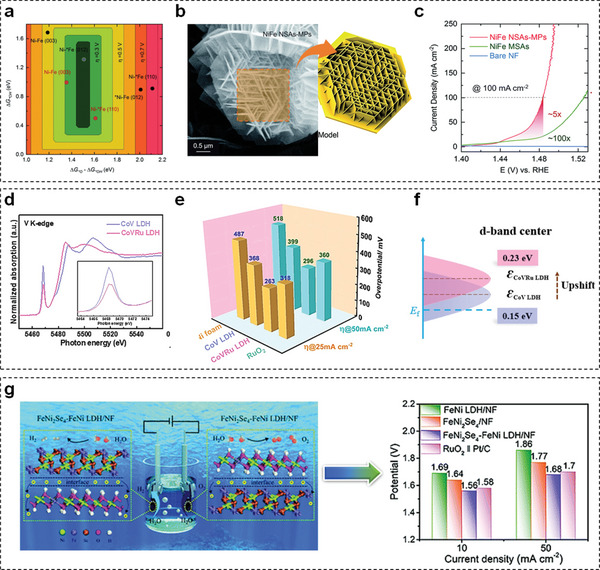
a) Theoretical overpotential plot for different crystal facets of NiFe‐LDHs. b) SEM image and corresponding schematic model of NiFe NSAs‐MPs. c) LSV curves of NiFe NSAs‐MPs, NiFe MSAs, and NF in 1 m KOH electrolyte at 5 mV s^−1^. Reproduced with permission.^[^ ^51^ ^]^ Copyright 2021, Royal Society of Chemistry. d) V K‐edge normalized XANES spectra of CoV‐LDHs and CoVRu‐LDHs nanosheets. e) Overpotential values of CoV‐LDHs, CoVRu‐LDHs, RuO_2_, and NF at 25 and 50 mA cm^−2^. f) d‐band center of CoV‐LDHs and CoVRu‐LDHs. Reproduced with permission.^[^ ^53^ ^]^ Copyright 2023, Elsevier. g) Cell voltage for a symmetrical FeNi_2_Se_4_‐FeNi‐LDHs‐based electrolyzer at current densities of 10 and 50 mA cm^−2^. Reproduced with permission.^[^ ^55^ ^]^ Copyright 2022, Royal Society of Chemistry.

Later on, versatile modification strategies including surface engineering, strain engineering, doping, and vacancy introduction are employed to ameliorate the O_2_ electrocatalytic evolution ability of TM‐LDHs nanosheets. Owing to the nearly identical length of Ni—O and Fe—O bonds, the synergistic interaction between Ni and Fe sites and high electronic conductivity,^[^ ^50^ ^]^ it is noticed that recent reported TM‐LDHs nanosheets for OER are mostly constructed on the basis of NiFe‐LDHs. For example, Zhao et al. found that (012) edge plane possessed the lowest onset overpotential for OER through theoretical calculation methods (**Figure** **7**a).^[^ ^51^ ^]^ According to the theoretical results, NiFe‐LDHs nanosheet‐arrays‐on‐microplates (NiFe NSAs‐MPs) with abundant exposed (012) crystal planes were fabricated and the corresponding scanning electron microscopy (SEM) image is shown in Figure 7b. As expected from linear sweep voltammetry (LSV) curves in Figure 7c, the overpotential of NiFe NSAs‐MPs at 100 mA cm^−2^ was only 250 mV, which was far below the overpotential of (003) plane‐dominated NiFe‐LDHs microsheet arrays (NiFe‐LDHs‐MSAs) and pure nickel foam (NF). Afterward, abundant dislocations are validated to promote the OER active phase formation of NiFe‐LDHs nanosheets.^[^ ^52^ ^]^ Compare with pristine NiFe‐LDHs nanosheets, the overpotential of NiFe‐LDHs nanosheets with ample dislocations at 10 mA cm^−2^ was reduced from 223 to 199 mV, manifesting the positive influence of dislocation on OER performance.

Doping and vacancy generation are common methods to tune the electronic structure of TM‐LDHs nanosheets at the atomic or molecular level, and thus modulate their inherent OER activity. For example, Zeng et al. successfully incorporated Ru atoms onto the surface of CoV‐LDHs nanosheets to enhance OER activity.^[^ ^53^ ^]^ According to X‐ray absorption near‐edge structure (XANES) results (Figure 7d), the addition of Ru^3+^ reduced the valence state of vanadium element to cause the local charge redistribution, which strengthened the electronic coupling between Ru atoms and CoV‐LDHs nanosheets. As a result, the CoVRu‐LDHs nanosheets only required an overpotential of 296 mV to gain a current density of 50 mA cm^−2^, while CoV‐LDHs and RuO_2_ required 395 and 353 mV, respectively (Figure 7e). Meanwhile, according to the d‐band center positions of CoV‐LDHs and CoVRu‐LDHs in Figure 7f, the upshift implied the existence of more unoccupied states, which was prone to capture electrons. Both experimental and theoretical results confirmed the beneficial effect of Ru doping on OER performance. Moreover, Xie et al. clarified the role of metal vacancies on OER activity.^[^ ^54^ ^]^ Concretely, after comparing free energy plots of NiFe‐LDHs nanosheets with M(II) and M(III) defects, the overpotential of Ni(II)‐Fe(III) sites was 0.2 eV lower than that of Ni(II)‐Ni(II) sites, denoting that M(II) defects were more effective during OER process.

To accelerate the kinetic rate of OER, TM‐LDHs nanosheets are always coupled with other materials, such as carbon materials. It is found that Co‐C@NiFe‐LDHs nanofibrous electrocatalyst exhibited better electrocatalytic activity than and expensive RuO_2_, which was ascribed to the fast charge transfer between NiFe‐LDH nanosheets and Co–C.^[^ ^56^ ^]^ Furthermore, heterogenous interface engineering on TM‐LDHs nanosheets has been also broadly explored to improve their OER activity. For example, Ouyang et al. combined p‐type CuO nanowires with n‐type FeCoNi‐LDHs nanosheets to construct heterostructured electrocatalyst for OER.^[^ ^57^ ^]^ Owing to the difference of Fermi levels between FeCoNi‐LDHs and CuO, an electric field was generated at the heterogenous interface to induce charge transfer within the whole electrocatalyst and thereby promote the adsorption ability of OH^−^, giving rise to elevated OER activity. Yu et al. fabricated FeNi_2_Se_4_‐FeNi‐LDHs bifunctional electrocatalyst for water splitting via partial selenization of FeNi LDHs nanosheets.^[^ ^55^ ^]^ Similarly, coupling counterparts in FeNi_2_Se_4_‐FeNi‐LDHs optimized the electronic environment around active center to enhance their electrocatalytic activities. Therefore, a symmetrical FeNi_2_Se_4_‐FeNi‐LDHs‐based electrolyzer could deliver 10 mA cm^−2^ by applying a cell voltage of 1.56 V (Figure 7g).

According to **Table** **1**, all TM‐LDHs nanosheets exhibit high OER activity in alkaline electrolyte. At present, the OER mechanism of most TM‐LDHs nanosheets follows AEM pathway, while LOM pathway is appropriate for TM‐LDHs nanosheets with modulated lattice oxygen coordination environment.^[^ ^79^, ^94^ ^]^ In a practical OER process, the low intrinsic diffusivity of activated lattice oxygen from bulk to surface could lead to the alternative mechanistic transformation between LOM and AEM, resulting in insufficient utilization of active sites.^[^ ^48^ ^]^ Thus, the difference in OER performance between AEM‐ and LOM‐based TM‐LDHs nanosheets is indistinct. With this consideration, the morphology of OER electrocatalysts should be designed to optimize their electronic/ionic transfer ability. Moreover, in order to further enhance the intrinsic OER activity of TM‐LDHs nanosheets, novel mechanism is still needed to completely break the scaling relationship between OH* and OOH* intermediates.

**Table 1:** Recent reported TM‐LDHs nanosheets for OER

Materials	Electrolyte	*η* [mV @ mA cm^−2^]	Tafel slope [mV dec^−1^]	Refs.
CoV‐LDHs	1 m KOH	250 @ 10	44	[58]
NiFe‐LDHs	1 m KOH	270 @ 10	48.6	[59]
NiFe‐LDHs‐MPs	1 m KOH	250 @ 100	34.5	[51]
NiFe‐LDHs	1 m KOH	324 @ 10	57.4	[60]
CoNi‐LDHs‐E	1 m KOH	280 @ 10	81	[61]
Ta‐NiFe LDHs	1 m KOH	260 @ 50	58.95	[62]
D‐NiFe LDHs	1 m KOH	199 @ 10	26.9	[52]
NiFe LDHs/Co_1−_ *_x_* S	1 m KOH	251 @ 10	41.67	[63]
NiFeV‐LDHs	1 m KOH	195 @ 20	42	[64]
NiFe_III_(1:1)‐LDHs	1 m KOH	183 @ 10	31.1	[65]
NiCo_1_Fe_1_‐LDHs	1 m KOH	231 @ 10	59	[66]
CoVRu‐LDHs	1 m KOH	263 @ 25	74.5	[53]
Fe‐NiV‐LDHs	1 m KOH	255 @ 10	56	[67]
NiFeV‐LDHs	1 m KOH	224 @ 10	32.7	[68]
NiFeNb‐0.25‐LDHs	1 m KOH	277 @ 100	50.6	[69]
NiFeCo‐LDHs	1 m KOH	249 @ 10	42	[70]
v‐NiFe‐LDHs	1 m KOH	195 @ 10	47.9	[71]
EE‐NiFe‐LDHs	1 m KOH	205 @ 10	41.8	[72]
NiCoFe‐LDHs	1 m KOH	174 @ 10	50	[73]
NiFe‐LDHs‐V_Ni_	1 M KOH	229 @ 10	62.9	[74]
v‐NiFe‐LDHs	1 m KOH	150 @ 10	37.1	[75]
D‐NiFeZn‐LDHs	0.1 m KOH	200 @ 20	34.9	[54]
NiFe‐LDHS‐V_O_	1 m KOH	230 @ 10	39.6	[76]
D‐CoFe‐LDHs	1 m KOH	283 @ 10	39	[77]
MnNiFe‐LDHs‐laser	1 m KOH	220 @ 10	37	[78]
M‐NiFe‐LDHs	1 m KOH	217 @ 10	45.1	[79]
AGC/MnCo‐LDHs	1 m KOH	370 @ 10	127.5	[80]
Co‐C@NiFe‐LDHs	1 m KOH	249 @ 10	57.9	[56]
FeNi‐LDHs/CoP	1 m KOH	231@ 20	33.5	[81]
FeCoNi‐LDHs/CuO/Cu	1 m KOH	243.1 @ 50	63.8	[57]
FeNi_2_Se_4_‐FeNi‐LDHs	1 m KOH	205 @ 10	30.14	[55]
MIM‐CoFe‐LDHs	1 m KOH	216.8@10	39.3	[82]
CoNi‐LDHs/Ti_3_C_2_T*_x_*	1 m KOH	200 @ 50	68	[83]
CoNi‐LDHs@PCPs	1 m KOH	350 @ 10	58	[84]
Ni_3_S_2_/Cu‐NiCo‐LDHs	1 m KOH	119 @ 10	70	[85]
NiCo‐LDHs/NiCoS	1 m KOH	308@ 100	48	[86]
A‐NiFe‐LDHs/CuS	1 m KOH	240 @ 100	41	[87]
NiCo@NiFe‐LDHs	1 m KOH	184 @ 10	46.1	[88]
Fe_2_O_3_/NiFe‐LDHs	1 m KOH	220 @ 500	32	[9b]
NiFe‐LDH/V‐Co_4_N	1 m KOH	203 @ 10	26	[89]
Mo‐NiS*_x_* @NiFe‐LDHs	1 m KOH	271 @ 100	44.41	[90]
NF‐Ni_2_P@NiFe LDHs	1 m KOH	203.5 @ 10	48.29	[91]
NiCo_2_S_4_@NiFe‐LDHs	1 m KOH	287 @ 10	86.4	[92]
Ni_3_S_2_/VG@NiCo‐LDHs	1 m KOH	350 @ 100	65	[93]

### 3.2 Hydrogen Evolution Reaction

#### 3.2.1 Mechanism

Hydrogen has been considered as an ideal fuel because of its extremely high heating value (142 MJ kg^−1^), earth abundance, and environmental benignity.^[^ ^95^ ^]^ In natural environment, water and hydrocarbon are two primary sources for hydrogen. Currently, industrial hydrogen production mainly relies on steam reforming of hydrocarbons, which consumes fossil fuels and release plenty greenhouse gas.^[^ ^96^ ^]^ In contrast, water electrolysis with zero emission and high hydrogen purity is regard as a more promising approach.^[^ ^97^ ^]^

**Figure 8:**
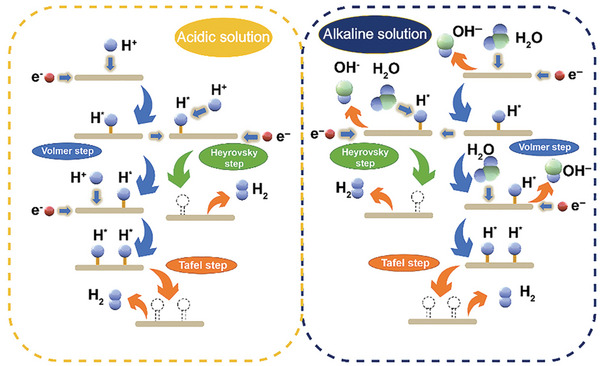
The schematic illustration of HER mechanism in acidic and alkaline media. Reproduced with permission.^[^ ^98b^ ^]^ Copyright 2022, Wiley.

HER is the cathodic reaction of water electrolysis, which involves two‐electron transfer. Since HER performance is pertinent on the pH value of electrolyte, the mechanism of HER needs to be elucidated in different electrolytes (**Figure** **8**).^[^ ^98^ ^]^ In acidic medium, a proton is initially adsorbed on active sites to form H* (Volmer step). Subsequently, H* is combined with either another H* (Tafel step) or another proton (Heyrovsky step) to release hydrogen. Generally, the Tafel slope value is a classical indicator to determine rate‐demining step (RDS) in HER.^[^ ^99^ ^]^ For Volmer, Tafel, and Heyrosky steps, the corresponding Tafel slope values are estimated as 120, 30, and 40 mV dec^−1^.^[^ ^100^ ^]^ According to these values, the mechanism of HER can be separated into Volmer–Tafel and Volmer–Heyrosky mechanism. The reaction pathway in neutral or alkaline medium is nearly identical to that in acidic medium except that water molecules are participated to provide protons. However, because of the additional water dissociation step, the HER process in neutral and alkaline electrolytes is reported as two to three order of magnitude slower than that in acidic electrolyte.^[^ ^101^ ^]^ Consequently, both water dissociation and hydrogen adsorption should be considered in designing efficient electrocatalysts toward neutral and alkaline HER.

#### 3.2.2 Research

Over the past few decades, remarkable endeavors have been devoted to construct nonprecious TM‐LDHs nanosheets to replace noble metal‐based electrocatalysts. However, HER performance of pristine TM‐LDHs nanosheets is not desirable in alkaline electrolyte owing to the sluggish kinetics of Volmer step. Thus, various strategies have been utilized to improve their HER performance.

**Figure 9:**
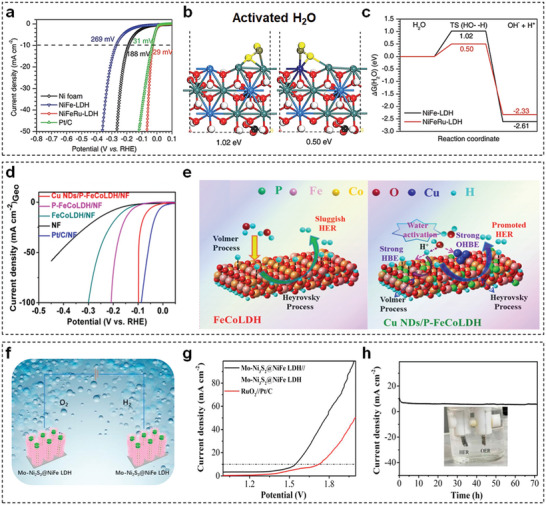
a) LSV curves of NiFeRu‐LDHs, NiFe‐LDHs, NF, and Pt/C electrocatalysts for HER. b) Adsorption free energies of activated H_2_O and c) corresponding free energy diagrams of NiFe‐LDHs and NiFeRu‐LDHs nanosheets. Reproduced with permission.^[^ ^102^ ^]^ Copyright 2018, Wiley. d) LSV curves of Cu NDs/P‐FeCo‐LDHs/NF, P‐FeCo‐LDHs/NF, FeCo‐LDHs/NF, Pt/C/NF, and NF with a scan rate of 5 mV s^−1^ for HER. e) Schematic representation of alkaline HER mechanism on FeCo‐LDHs and Cu NDs/P‐FeCo‐LDHs. Reproduced with permission.^[^ ^105^ ^]^ Copyright 2021, Elsevier. f) Schematic of Mo‐Ni_2_S_3_@NiFe‐LDHs‐based symmetrical electrolyzer for water splitting. g) LSV curves of the symmetrical electrolyzer and RuO_2_//Pt/C couple. h) Stability test of the symmetrical electrolyzer over 72 h. Reproduced with permission.^[^ ^106^ ^]^ Copyright 2021, Elsevier.

HER activity of TM‐LDHs nanosheets could be enhanced through defect engineering, such as doping metal elements and constructing oxygen vacancies. Chen et al. doped noble metal Ru into NiFe‐LDHs (NiFeRu‐LDHs) nanosheets to achieve a superior HER performance.^[^ ^102^ ^]^ As shown in **Figure** **9**a, overpotential of NiFeRu‐LDHs nanosheets for HER was only 29 mV to drive a current density of 10 mA cm^−2^. Afterward, the superior HER activity was elucidated by density functional theory (DFT) calculations (Figure 9b,c). After Ru doping, energy barrier of the Volmer step was decreased from 1.02 to 0.5 eV, suggesting that the HER kinetic process was greatly accelerated. The beneficial effect of Ru doping was also validated on NiV‐LDHs nanosheets.^[^ ^103^ ^]^ Theoretical calculations suggested that Ru doping could significantly decrease the hydrogen adsorption energy in both Volmer and Heyrovsky steps, thus boosting the HER kinetics. Furthermore, Liu et al. synthesized Ce‐doped CoFe‐LDHs nanosheets with abundant oxygen vacancies (V‐Ce/CoFe‐LDHs) via a plasma etching strategy.^[^ ^104^ ^]^ Herein, Ce doping and oxygen vacancy not only lowered the energy barrier of HER, but also led to more active sites and improved electrical conductivity, thus giving rise to an excellent HER performance.

Hybridization of TM‐LDH materials with highly conductive materials is another strategy to expedite HER kinetics. Taking graphene oxide (GO) as example, the appropriate quantity of GO can control the growth of NiFe‐LDHs nanosheets during the electrodeposition process.^[^ ^107^ ^]^ The as‐synthesized composites possessed a large electrochemical surface area, strong electronic interaction between NiFe‐LDHs and GO, which are advantageous for hydrogen generation. In addition, Feng et al. employed P doping and subsequent Cu nanodots (NDs) deposition to delicately construct efficient Cu NDs/P‐FeCo‐LDHs electrocatalyst for HER.^[^ ^105^ ^]^ The Cu NDs/P‐FeCoLDHs exhibited Pt‐like HER activity with an overpotential of 63 mV at 10 mA cm^−2^ (Figure 9d), which is much lower than those of their counterparts. According to mechanistic analysis, P doping and deposited Cu NDs were responsible for optimizing H* binding energy and accelerating water dissociation on the surface of electrocatalyst, respectively. Thus, the combined method can effectively expedite the kinetics of alkaline HER (Figure 9e).

Furthermore, TM‐LDHs‐based hybrid electrocatalysts have been used in a practical electrolyzer with high H_2_ production efficiency. As shown in Figure 9f, heterostructured Mo‐Ni_2_S_3_@NiFe‐LDHs electrocatalyst was utilized in a symmetrical alkaline electrocatalytic cell for water dissociation.^[^ ^106^ ^]^ Surprisingly, in comparison with Pt/C//RuO_2_, the Mo‐Ni_2_S_3_@NiFe‐LDHs‐based electrolyzer only needed 1.54 V to accomplish 10 mA cm^−2^ (Figure 9g). Also, the symmetric electrolyzer exhibited fabulous durability for 72 h (Figure 9h).

As summarized in **Table** **2**, most TM‐LDH nanosheets present high HER efficiency in alkaline electrolyte. According to their Tafel slope values, the desorption of hydrogen gas in Tafel and Heyrosky steps are determined as main RDS for alkaline HER. Thus, the modulation of H* desorption energy is crucial for designing TM‐LDHs nanosheets as high‐performance HER electrocatalysts. Besides, long‐term stability of TM‐LDHs nanosheets at a large current density is still far from satisfaction. Combining the state‐of‐art fabrication methods, the construction of TM‐LDHs nanosheet‐based hybrid structure with optimal hydrogen adsorption/desorption ability is expected to realize long‐term efficient hydrogen production.

**Table 2:** Recent reported TM‐LDHs nanosheets for HER

Materials	Electrolyte	*η* [mV @ mA cm^−2^]	Tafel slope [mV dec^−1^]	Refs.
NiFeRu‐LDHs	1 m KOH	29 @ 10	31	[102]
NiMoCo‐LDHs	1 m KOH	123 @ 10	67.39	[108]
NiVRu‐LDHs	1 m KOH	12 @ 10	40	[103]
NiFeAu‐LDHs	1 m KOH	89 @ 10	90	[109]
La‐NiFe LDHs‐3	1 m KOH	57 @ 10	73.7	[110]
Ir–NiCo LDHs	1 m KOH	21 @ 10	33.2	[111]
Pt@CuFe‐LDHs	1 m KOH	33 @ 10	34	[112]
V‐Ce/CoFe‐LDHs	1 m KOH	73 @ 10	69	[104]
A‐NiCo‐LDHs/NF	1 m KOH	151 @ 100	57	[113]
CoFe‐LDHs‐F	1 m KOH	166 @ 10	92	[114]
Co_3_Fe_1_‐LDHs/rGO	1 m KOH	110 @ 10	106	[115]
GO‐NiFe‐LDHs	1 m KOH	119 @ 10	36	[107]
H‐CoS*_x_* @NiFe LDHs	1 m KOH	95 @ 10	90	[116]
Ni_3_Se_4_@NiFe LDHs	1 m KOH	85 @ 10	98.6	[117]
Cu@NiFe‐LDHs	1 m KOH	116 @ 10	58.9	[118]
Ag@NiFe‐LDHs	1 m KOH	78 @ 10	58.1	[119]
Cu NDs/P‐FeCo‐LDHs	1 m KOH	63 @ 10	41.74	[105]
Mo‐Ni_2_S_3_@NiFe LDHs	1 m KOH	109 @ 10	36.5	[106]

## 4 Urea Oxidation/Nitrogen Reduction Reaction

### 4.1 Urea Oxidation Reaction

#### 4.1.1 Mechanism

Urea electrolysis (CO(NH_2_)_2_ + H_2_O → N_2_ + 3H_2_ + CO_2_) is promising to replace conventional water electrolysis for hydrogen production due to its low theoretical electrocatalytic voltage (0.37 V vs 1.23 V) and abundant natural sources.^[^ ^120^ ^]^ UOR is an anodic reaction with sluggish six‐electron transfer, which determines the overall electrocatalytic performance.^[^ ^121^ ^]^ Generally, UOR is favorable to proceed in alkaline electrolyte since neutral and acid electrolytes can accelerate the corrosion of metallic electrocatalysts.^[^ ^122^ ^]^

**Figure 10:**
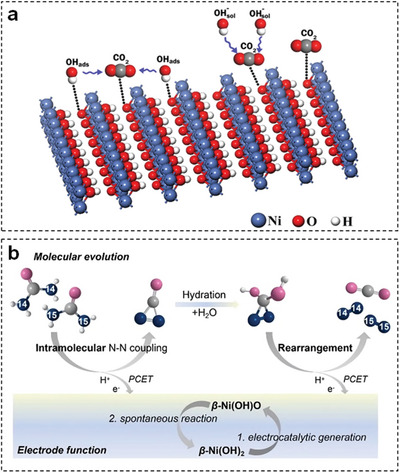
a) Schematic illustration of indirect UOR mechanism. Reproduced with permission.^[^ ^123^ ^a]^ Copyright 2016, Elsevier. b) The representation of intramolecular N─N coupling mechanism for UOR. Reproduced with permission.^[^ ^127^ ^]^ Copyright 2021, Wiley.

For the alkaline UOR, nickel‐based electrocatalysts have been mostly reported due to their low‐cost and intriguing UOR performance. Initially, NiOOH was considered as active species of nickel‐based electrocatalysts and two possible mechanisms were proposed: 1) direct oxidation mechanism [NiOOH·CO(NH_2_)_2_]_ads_ + 6OH^−^ → [NiOOH·CO_2_]_ads_ + N_2_ + 5H_2_O + 6e^−^; 2) indirect oxidation mechanism 6NiOOH + CO(NH_2_)_2_ + H_2_O → 6Ni(OH)_2_ + N_2_ + CO_2_ (**Figure** **10**a).^[^ ^123^ ^]^ Afterward, the assumptions were investigated by in situ tools. Vedharathinam et al. utilized in situ time‐resolved Raman spectroscopy to study the phase transformation of NiOOH during the alkaline UOR process under open‐circuit conditions.^[^ ^124^ ^]^ In pure alkaline electrolyte, two characteristic peaks of NiOOH at 479/559 cm^−1^ remained unchanged for 13 min. With the addition of urea, a new peak of Ni(OH)_2_ at 528 cm^−1^ was detected after 4 min. These comparative results indicated that NiOOH could be reduced to Ni(OH)_2_ during alkaline UOR, which was also verified by in situ X‐ray diffraction technique.^[^ ^125^ ^]^ Therefore, the mechanism of alkaline UOR is confirmed as indirect catalyst regeneration mechanism. To better understand alkaline UOR, Daramola et al. calculated three possible reaction pathways of urea dissociation with NiOOH using DFT calculations.^[^ ^126^ ^]^ After analyzing rate constants and free energies of intermediate steps, the optimized pathway was proposed as M·CO(NH_2_)_2_ → M·CO(NH·NH_2_) → M·CO(NH·NH) → M·CO(NH·N) → M·CO(N_2_) → M·CO(OH) → M·CO(OH·OH) → M·COO (M = NiOOH) and the last step of CO_2_ desorption was found to be the RDS.

Recently, Chen et al. rebuilt the conventional indirect mechanism of UOR based on detailed experimental and theoretical results.^[^ ^127^ ^]^ As seen in Figure 10b, *β*‐Ni(OH)_2_ is electrocatalytic dehydrogenated to generate *β*‐Ni(OH)O intermediates, which could further react with urea via a spontaneous dehydrogenative oxidation process. For the second spontaneous reaction, *β*‐Ni(OH)O intermediates initially seize the protons from two amine groups in one urea molecule and CO(NN) intermediates with an intramolecular N—N bond are formed through proton‐coupled electron transfer (PCET). After complicated hydration, rearrangement, and PCET processes, CO(NN) intermediates are transformed into N_2_ and CO_2_. In sum, current understanding of UOR is limited and great efforts should be devoted to clarifying the UOR mechanism by employing advanced in situ characterization instruments and powerful theoretical calculations.

#### 4.1.2 Research

**Figure 11:**
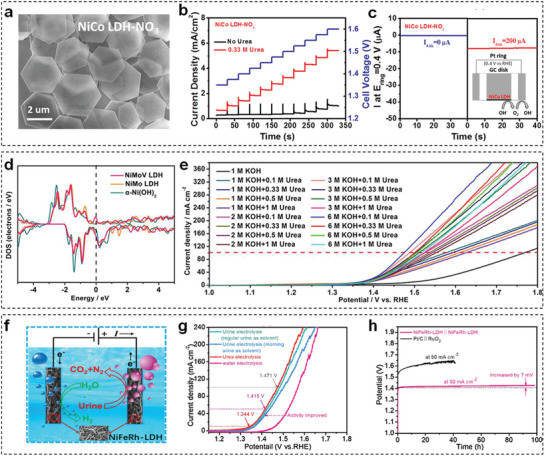
a) SEM images of nitrate‐exchanged NiCo‐LDHs nanosheets. b) Voltage step analyses in 1 m KOH with and without 0.33 M urea. c) The ring current on RRDE in 1 m KOH + 0.33 m urea electrolyte with the ring potential of 0.4 V versus RHE. Reproduced with permission.^[^ ^128^ ^]^ Copyright 2019, American Chemical Society. d) Calculated total DOS of NiMoV‐LDHs/NF, NiMo‐LDHs/NF, and *α*‐Ni(OH)_2_/NF. e) Polarization curves of a NiMoV‐LDHs/NF‐based urea electrolytic cell in different electrolytes. Reproduced with permission.^[^ ^130^ ^]^ Copyright 2022, Elsevier. f) Schematic diagram of a symmetrical urea electrolytic cell using NiFeRh‐LDHs nanosheets. g) Comparison of CV curves for water, urea, and urine electrolysis. h) Stability measurement of NiFeRh‐LDHs nanosheets for urea electrolysis at 50 mA cm^−2^. Reproduced with permission.^[^ ^132^ ^]^ Copyright 2021, Elsevier.

TM‐LDHs nanosheets have intensively emerged as electrocatalysts for UOR. Specifically, Zeng et al. employed a scalable liquid exfoliation method to obtain nitrate‐exchanged NiCo‐LDHs nanosheets for efficient UOR.^[^ ^128^ ^]^ The synthetic method with long‐term anion exchange process generated NiCo‐LDHs nanosheets with well‐defined hexagonal shape (**Figure** **11**a). In comparison with carbonate‐exchanged NiCo‐LDHs and NiCo hydroxide, nitrate‐exchanged NiCo‐LDHs nanosheets displayed better UOR performance due to larger interlayer spacing. Moreover, the current efficiencies of OER and UOR were estimated via voltage step analyses and rotating ring‐disk electrode (RRDE) tests. In the voltage step analyses, the cell voltage range was applied between NiCo‐LDHs nanosheets and Pt foil from 1.35 to 1.60 V. As seen in Figure 11b, the current efficiencies of UOR were >54% at all cell voltage values and achieved a maximal value of 88% at 1.50 V. In RRDE tests, a ring current of 7.6 µA was detected under the constant disk current of 200 µA in alkaline electrolyte with urea (Figure 11c). The corresponding current efficiency of UOR was calculated as around 80%, which is consistent with voltage step analyses. Wang et al. successfully applied a facile hydrothermal method to grow Cr‐doped CoFe‐LDHs (CoFeCr‐LDHs) nanosheets on NF substrate, which possessed an ultralow potential of 1.305 V versus reversible hydrogen electrode (RHE) for UOR at a current density of 10 mA cm^−2^.^[^ ^129^ ^]^ The outstanding UOR performance was considered to be originated from the inherent electron‐withdrawing ability of Cr ions. Besides, a full electrolyzer was assembled with CoFeCr‐LDHs nanosheets anode and Pt‐C cathode to generate hydrogen. With the aid of urea, the cell voltage of the electrolyzer to achieve a current density of 10 mA cm^−2^ was decreased from 1.467 to 1.329 V. Thus, efficient hydrogen production could be realized by driving such a device with a commercial 1.5 V battery.

Based on above experimental results, doping high‐valence metal elements into TM‐LDHs nanosheets is validated as an effective strategy to obtain superior UOR performance. Meanwhile, the beneficial effect of doping is also studied by many researchers in theoretical perspective. For example, Wang et al. designed Mo and V co‐doped Ni‐based LDHs (NiMoV‐LDHs) nanosheets as highly efficient electrocatalysts for UOR and investigated the effect of dopants through DFT calculations.^[^ ^130^ ^]^ Based on the total density of states (DOS) curves in Figure 11d, NiMoV‐LDHs possessed the strongest DOS signal around the Fermi level, denoting that Mo and V‐doping could significantly promote the electron transfer during UOR. Therefore, a urea electrolytic cell based on NiMoV‐LDHs nanosheets showed superior UOR performance in various simulated urea‐polluted electrolytes (Figure 11e). Except for doping, incorporating highly conductive carbon materials with TM‐LDHs nanosheets is another strategy to improve UOR performance. For instance, Feng et al. constructed a novel hybrid UOR electrocatalyst by anchoring fullerene quantum dot decorated CoNi‐LDHs nanosheets on porous NF substrate (FQD/CoNi‐LDHs/NF), which required 1.42 V versus RHE to deliver a current density of 100 mA cm^−2^ for UOR.^[^ ^131^ ^]^ Subsequently, the vital role of FQD was revealed by performing DFT calculations on CoNi‐LDHs and FQD/CoNi‐LDHs. After comparing the DOS profiles of these electrocatalysts, it is clearly concluded that FQD could increase the electronic conductivity and charge density, resulting in fascinating UOR performance of FQD/CoNi‐LDHs/NF.

To further extend the application of TM‐LDHs nanosheets in UOR, Sun et al. fabricated bifunctional Rh‐doped NiFe‐LDHs (NiFeRh‐LDHs) nanosheets via an ethylene glycol‐assisted hydrothermal method.^[^ ^132^ ^]^ The introduction of Rh‐doping optimized adsorption energy of H* and urea, which boosted HER and UOR performance of NiFeRh‐LDHs nanosheets. Thus, NiFeRh‐LDHs nanosheets could be applied as high‐performance anode and cathode simultaneously in a symmetrical urea electrolytic cell (Figure 11f). Figure 11g presents the comparison of cyclic voltammetry (CV) curves for water, urea, and urine electrolysis using this novel designed cell. It is obviously seen that the electrolytic performance of urea and urine surpassed that of water significantly. Specifically, the cell voltages for urea electrolysis were required as 1.344 and 1.471 V to afford current densities of 10 and 100 mA cm^−2^, respectively. Furthermore, the symmetric electrolytic cell could operate steadily at 50 mA cm^−2^ for at least 100 h (Figure 11h), demonstrating excellent stability for urea electrolysis. Up to date, although significant progress has been made on designing novel TM‐LDHs nanosheets for UOR, the lowest overpotential value in recent reported publications is still larger than 900 mV at 10 mA cm^−2^ (**Table** **3**). Strategies to reduce the energy barrier of the *COO desorption step are required for accelerating UOR kinetics on TM‐LDHs nanosheets. Therefore, new mechanisms and reaction pathways are urgently demanded to provide a guideline for constructing TM‐LDHs nanosheets with optimal UOR performance.

**Table 3:** Recent reported TM‐LDHs nanosheets for UOR

Materials	Electrolyte	Potential [V vs RHE]	Current density [mA cm^−2^]	Tafel [mV dec^−1^]	Refs.
CoFeCr‐LDHs	0.33 m urea	1.305	10	85	[129]
NiMoV‐LDHs	0.33 m urea	1.40	100	24.29	[130]
FQD/CoNi‐LDHs	0.5 m urea	1.36	10	17	[131]
NiMn‐LDHs	0.5 m urea	1.351	20	38.9	[133]
NiCo‐LDHs	0.33 m urea	1.36	50	45	[134]
pa‐NiFe‐LDHs NS	0.33 m urea	1.362	30	33	[135]
Cu‐Ni_3_S_2_@NiFe‐LDHs‐200	0.5 m urea	1.319	100	37.78	[136]
NiFe‐LDHs‐F‐4	0.33 m urea	1.44	50	31	[137]
NiAl‐LDHs	0.33 m urea	1.42	10	59.8	[30]
CoNi‐LDHs‐3	0.33 m urea	1.39	100	16	[138]
NiFe‐LDHs/EG	0.33 m urea	1.34	50	44	[139]
Ce(OH)_3_@NiFe‐LDHs	0.5 m urea	1.40	10	30.3	[140]
H‐NiFe‐LDHs	0.33 m urea	1.397	10	41.7	[141]
MoS_2_/Ni_3_S_2_/NiFe‐LDHs	0.5 m urea	1.396	100	36	[142]
NiCo‐LDHs‐[MoS_4_]^2−^	0.33 m urea	1.34	10	29	[143]
CoMn‐LDHs	0.33 m urea	1.326	10	73	[144]

### 4.2 Nitrogen Reduction Reaction

#### 4.2.1 Mechanism

Ammonia (NH_3_) is not only widely used in the synthesis of fertilizers, drugs, and chemicals, but also considered as an alternative fuel to store hydrogen.^[^ ^145^ ^]^ Currently, the majority of NH_3_ is produced through the conventional Haber–Bosch process at both high temperature and high pressure.^[^ ^146^ ^]^ Under this circumstance, a possible alternative to the Haber–Bosch process is electrocatalytic NRR under mild conditions.^[^ ^147^ ^]^ In acidic and alkaline environment, reaction equations of NRR are expressed as N_2_ + 6H^+^ + 6e^−^ → 2NH_3_ and N_2_ + 6H_2_O + 6e^−^ → 2NH_3_ + 6OH^−^, respectively.^[^ ^148^ ^]^

**Figure 12:**
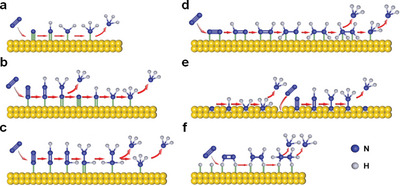
Schematic illustration of possible pathways for electrocatalytic conversion of N_2_ to NH_3_. Reproduced with permission.^[^ ^149^ ^]^ Copyright 2022, Wiley.

Generally, NRR occurs through N_2_ adsorption, hydrogenation, and subsequent NH_3_ desorption.^[^ ^149^ ^]^ Based on the order of cleavage and hydrogenation processes, dissociative and associative mechanism have been proposed.^[^ ^150^ ^]^ For dissociative mechanism, the strong N≡N is cleaved prior to hydrogenation (**Figure** **12**a). Because of extremely high bonding energy of N≡N, dissociative mechanism is mainly applicable for the industrial Haber–Bosch process with harsh reaction conditions. For associative mechanism, the cleavage and hydrogenation processes are preceded simultaneously. Considering the possible adsorption modes and hydrogenation sequences of nitrogen atoms, the associative mechanism is further classified into distal, alternating, and enzymatic pathways.^[^ ^151^ ^]^ Figure 12b,c depicts the distal and alternating pathways. For both pathways, N_2_ is initially end‐on adsorbed on the catalyst surface. Afterward, in the distal pathway, the nitrogen atom far away from the catalyst surface undergoes a preferential hydrogenation to release the first NH_3_ molecule, and the remained nitrogen atom follows the same procedure to generate the second NH_3_ molecule. Conversely, in the alternating pathway, two nitrogen atoms are hydrogenated alternately to produce NH_3_ molecules after end‐on adsorption on the catalyst surface. Lastly, in the enzymatic pathway (Figure 12d), two nitrogen atoms are first connected to the catalyst surface through side‐on adsorption, and the subsequent hydrogenation process is same as the alternating pathway.

Recently, novel mechanisms including Mars–van Krevelen (MvK) pathway^[^ ^152^ ^]^ and surface‐hydrogenation pathway^[^ ^153^ ^]^ have been proposed through DFT calculations. Concretely, these two pathways are suitable for explaining NRR on the surface of transition metal nitrides and noble metals, respectively. In the MvK pathway (Figure 12e), lattice nitrogen atoms are first hydrogenated on the catalyst surface. Then, the newly supplemented N_2_ are adsorbed to these resulting nitrogen vacancies and further hydrogenated to release NH_3_. In the surface‐hydrogenation pathway (Figure 12f), the potential‐dependent Volmer reaction initially occurs to generate H* on the catalyst surface. Subsequently, surface H* activates N_2_ to produce N_2_H_2_* with relatively high energy input. Finally, NH_3_ is released by spontaneous reduction of N_2_H_2_*. Although new mechanistic pathways have validated to decrease the onset potential of NRR significantly, it is inevitable to ignore numerous practical factors (e.g., pH value) by relying on theoretical models.^[^ ^154^ ^]^ Therefore, the development of in situ characterization methods is essential to define the NRR process accurately.

#### 4.2.2 Research

Compared with UOR, the application of TM‐LDHs nanosheets for NRR has been less investigated. Zou et al. reported an efficient NRR electrocatalyst with the cross‐link of NiCo‐LDHs nanosheets.^[^ ^155^ ^]^ By adjusting the Ni/Co ratio, the highest NH_3_ yield was achieved as 52.8 µg h^−1^ mg_cat_^−2^. The importance of Ni/Co ratio was further studied by in situ Raman measurements and DFT calculations. After comparing the activation energies of NRR process on four possible reaction sites, Co‐Ni‐Ni was proposed as the optimized active center for NH_3_ production. Nevertheless, pristine TM‐LDHs nanosheets are still subjected to low conductivity, leading to sluggish N_2_ activated/dissociated steps.

**Figure 13:**
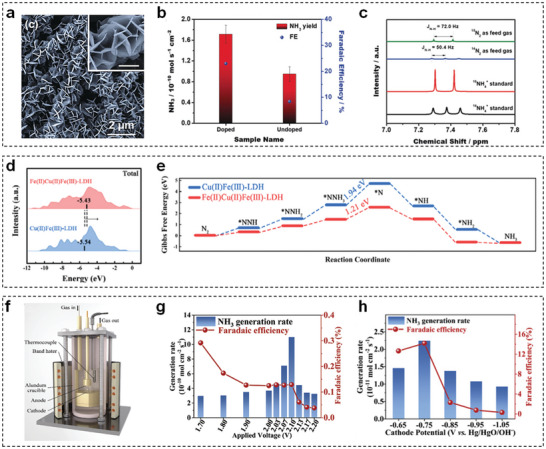
a) SEM image of P‐doped FeNi‐LDHs nanosheets. b) The comparison of NH_3_ yields and FEs of undoped and P‐doped FeNi‐LDHs nanosheets at −0.5 V versus RHE. c) ^1^H NMR spectra of electrolytes with ^15^N_2_ and ^14^N_2_ as feed gas. Reproduced with permission.^[^ ^156^ ^]^ Copyright 2020, Wiley. d) The calculated d‐band center of total elements and e) NRR free‐energy diagram for CuFe‐LDHs and Fe(II)Cu(II)Fe(III)‐LDHs nanosheets. Reproduced with permission.^[^ ^157^ ^]^ Copyright 2022, Wiley. f) Schematic diagram of the setup for single‐chamber cell. NH_3_ generation rates and FEs of the CoFe‐LDHs nanosheets using g) single‐chamber cell and h) H‐cell. Reproduced with permission.^[^ ^158^ ^]^ Copyright 2020, Elsevier.

Recently, several strategies have been validated to elevate NRR activity of TM‐LDHs nanosheets, including doping heteroatoms (e.g., P and Se) and introducing low‐valence transition metal ions. For example, Liu et al. coupled P‐doped FeNi‐LDHs nanosheet arrays with carbon nanofibers to achieve desired NRR performance.^[^ ^156^ ^]^ As seen in **Figure** **13**a, P‐doped FeNi‐LDHs nanosheets with the thickness of 20−30 nm were homogenously distributed on the surface of carbon nanofibers. Attributed to the generation of extra empty d orbitals from P‐doping, the maximum NH_3_ yield and corresponding Faradaic efficiency (FE) were increased from 0.95 × 10^−10^ to 1.7 × 10^−10^ mol s^−1^ cm^−2^ and from 8.4% to 23%, respectively (Figure 13b). Isotopic labeling experimental results in Figure 13c further demonstrated that the high selectivity toward NH_3_ was originated from the feed N_2_, not contaminants in the electrochemical testing system. Afterward, Kong et al. applied a modified hydrothermal method to bring low‐valence ferrous ion into CuFe‐LDHs (Fe(II)Cu(II)Fe(III)‐LDHs) nanosheets for effective NRR.^[^ ^157^ ^]^ After adding the ferrous ions, the d‐band center position was positively shifted from −5.54 to −5.43 eV (Figure 13d) and the energy barrier of potential‐determining step was decreased from 1.94 to 1.21 eV (Figure 13e), suggesting the enhanced N_2_ adsorption and reduction ability. As a result, Fe(II)Cu(II)Fe(III)‐LDHs nanosheets realized a decent NH_3_ yield rate of 33.1 ± 2.5 µg h^−1^ mg_cat_^−1^ with an FE of 21.7 ± 1.8% at −0.5 V versus RHE, which outperformed that of pristine CuFe‐LDHs nanosheets.

Apart from electronic structure modulation of TM‐LDHs nanosheets, developing hierarchical heterostructure is apt to accelerate NRR kinetics by exposing sufficient active sites and facilitating the electronic/ionic transport. As reported by Arif et al., the hierarchical CoVP@NiFeV‐LDHs heterostructure showed superior NRR performance over the individual components (CoVP, NiFeV‐LDHs nanosheets).^[^ ^159^ ^]^ Due to highly durable CoVP substrate, efficient pH‐universal NH_3_ generation was also realized on this dedicated designed NRR electrocatalyst. Furthermore, proper selection of reaction system is another key point for fully reflecting NRR performance of TM‐LDHs nanosheets. Qiao et al. evaluated NRR activities of CoFe‐LDHs nanosheets using a single‐chamber cell (Figure 13f) and a common H‐cell separately.^[^ ^158^ ^]^ Since HER and possible ammonia oxidation was inhibited to some extent in the H‐cell, the FE of CoFe‐LDHs nanosheets was elevated significantly after replacing single‐chamber cell by H‐cell (Figure 13g,h). Nevertheless, the corresponding NH_3_ generation capability in the H‐cell was decreased dramatically. Thus, the balance between the highest NH_3_ generation rate and FE should be carefully considered in the design of NRR experimental system. With tremendous efforts on TM‐LDHs nanosheets, both the NH_3_ generation rate and FE value have been greatly increased in the past few years. However, most recent reported TM‐LDHs nanosheets with undesired selectivity (**Table** **4**) cannot be compatible with the industrial requirements since the competing two‐electron HER is kinetically favored over six‐electron NRR.^[^ ^160^ ^]^ Meanwhile, the overall NH_3_ yield is also close related to the cleavage rate of strong N≡N on the surface of TM‐LDHs nanosheets. Therefore, developing advanced NRR system with the composition of novel TM‐LDHs nanosheets‐based electrocatalysts and nonaqueous electrolyte is expected to regulate the proton activity and realize efficient NH_3_ synthesis.

**Table 4:** Recent reported TM‐LDHs nanosheets for NRR

Materials	Electrolyte	FE	NH_3_ yield	Refs.
NiCo‐LDHs	0.1 m Na_2_SO_4_	11.5%	52.8 µg h^−1^ mg_cat_^−2^	[155]
P‐doped FeNi‐LDHs	0.1 m Na_2_SO_4_	23.0%	1.72 × 10^−10^ mol s^−1^ cm^−2^	[156]
Fe(II)Cu(II)Fe(III)‐LDHs	0.1 m Na_2_SO_4_	21.7%	33.1 µg h^−1^ mg_cat_^−2^	[157]
CoVP@NiFeV‐LDHs	0.05 m H_2_SO_4_	13.8%	1.6 µmol h^−1^ cm^−2^	[159]
CoFe‐LDHs	25.6 mol kg^−1^ KOH	0.129%	1.1 × 10^−9^ mol s^−1^ cm^−2^	[158]
Ni_0.75_Fe_0.25_Se_2_	0.1 m Li_2_SO_4_	12.3%	5.64 µg h^−1^ cm_cat_^−2^	[161]

## 5 Small Molecule Oxidation

### 5.1 Mechanism

Liquid small molecular fuels have been regarded as promising alternatives to gaseous hydrogen for fuel cells owing to their inexpensive price, high intrinsic energy density, and high safety.^[^ ^162^ ^]^ Understanding the mechanism of small organic molecule electro‐oxidation is essential for constructing efficient electrocatalysts. In this section, two representative reactions, methanol oxidation reaction (MOR) and ethanol oxidation reaction (EOR), are detailedly elucidated.

**Figure 14:**
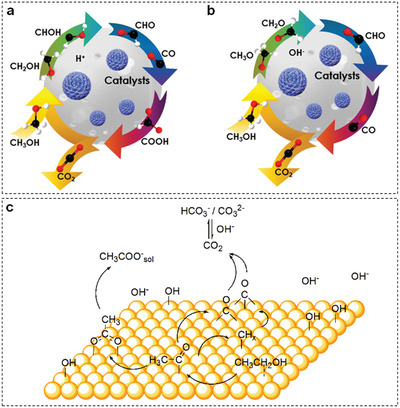
Schematic reaction pathways for MOR in a) acidic and b) alkaline electrolytes. Reproduced with permission.^[^ ^163^ ^]^ Copyright 2021, Wiley. c) Schematic reaction pathways for EOR in alkaline electrolyte. Reproduced with permission.^[^ ^167^ ^]^ Copyright 2014, American Chemical Society.

MOR is a six‐electron oxidation process to generate CO_2_ as final product via *CO and *CO‐free pathways. The *CO pathway in acidic and alkaline electrolytes is illustrated in **Figure** **14**a,b, respectively.^[^ ^163^ ^]^ In the acidic electrolyte, MOR undergoes the following steps: *CH_3_OH → *CH_2_OH → *CHOH → *CHO → *CO + H_2_O → *COOH → * + CO_2_.^[^ ^164^ ^]^ In the alkaline electrolyte, plenty OH^−^ are participated in dehydrogenation steps: *CH_3_OH → *CH_3_O → *CH_2_O → *CHO → *CO + *OH → * + CO_2_.^[^ ^165^ ^]^ It is widely accepted that the MOR performance in alkaline electrolyte is better than that in the acidic electrolyte since a high concentration of OH^−^ is beneficial for removing poisoning intermediate of *CO.^[^ ^165^ ^]^ Alternatively, the key intermediate *CHO is converted to *HCOOH instead of *CO in the *CO‐free pathway, and further hydrogenated into CO_2_.^[^ ^166^ ^]^

In comparison with MOR, the mechanism of EOR is more complicated. As displayed in Figure 14c, EOR proceeds through dual pathways, which include a complete oxidation pathway and a partial oxidation pathway.^[^ ^167^ ^]^ Concretely, the complete oxidation of ethanol leads to the production of CO_2_ (acidic) or HCO_3_^−^/CO_3_^2−^ (alkaline) via the cleavage of the C—C bond and multiple dehydrogenation steps. However, the intrinsic slow kinetics of C—C bond splitting gives rise to the unfavorable occurrence of the complete oxidation pathway.^[^ ^168^ ^]^ Thus, ethanol is prone to be partially oxidized to CH_3_COOH (acidic) or CH_3_COO^−^ (alkaline) via a four‐electron transfer process. At present, mechanism of MOR and EOR are mostly proposed based on the noble metal electrocatalysts, such as Pt and Pd.^[^ ^169^ ^]^ More attentions should be concentrated on the mechanistic study of earth‐abundant transition‐metal‐based electrocatalysts.

### 5.2 Research

**Figure 15:**
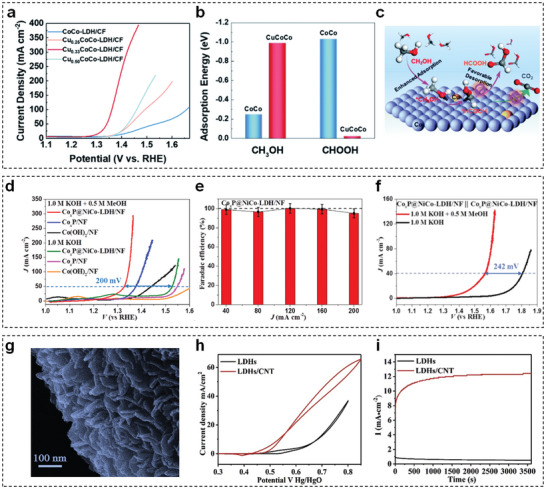
a) LSV curves of Cu*_x_* CoCo‐LDHs/CFs in 1 m KOH + 3 m CH_3_OH electrolyte. b) The calculated adsorption energy of CH_3_OH and formic acid molecules on CoCo‐LDHs and CuCoCo‐LDHs nanosheets. c) The schematic process of MOR on the surface of CuCoCo‐LDHs. Reproduced with permission.^[^ ^171^ ^]^ Copyright 2022, Royal Society of Chemistry. d) LSV curves of Co(OH)_2_, Co*_x_* P, and Co*_x_* P@NiCo‐LDHs toward MOR and OER. e) FE of formate generation at different current densities. f) LSV curves of Co*_x_* P@NiCo‐LDHs//Co*_x_* P@NiCo‐LDHs two‐electrode electrolyzer with and without CH_3_OH. Reproduced with permission.^[^ ^173^ ^]^ Copyright 2020, Elsevier. g) SEM image of NiFe‐LDHs/CNT spherical composite. h) CV curves of NiFe‐LDHs/CNT spherical composite and NiFe‐LDHs powder sample in 1 m KOH + 1 m CH_3_CH_2_OH electrolyte. i) Chronoamperometric curves of NiFe‐LDHs/CNT spherical composite and NiFe‐LDHs powder sample. Reproduced with permission.^[^ ^174^ ^]^ Copyright 2021, Elsevier.

Considering low cost, ample abundance, and good compatibility with alkaline electrolyte, TM‐LDHs nanosheets have drawn considerable attention as promising alternatives to traditional noble metal electrocatalysts in MOR and EOR. Initially, pristine TM‐LDHs nanosheets were used without any modification, such as NiCo‐LDHs^[^ ^170^ ^]^ and NiAl‐LDHs.^[^ ^18c^ ^]^ Afterward, the strategies related to the modulation of electronic configuration of TM‐LDHs nanosheets have been performed to optimize the methanol and ethanol adsorption ability, leading to enhanced intrinsic MOR and EOR activities. For instance, Liu et al. prepared hierarchical Cu*_x_* CoCo‐LDHs nanosheets through the pseudomorphic transformation of Cu*_x_* Co‐MOFs.^[^ ^171^ ^]^ Compared with the pristine CoCo‐LDHs, all Cu*_x_* CoCo‐LDHs showed higher MOR activity and the lowest potential was achieved as 1.34 V versus RHE to reach 50 mA cm^−2^ (**Figure** **15**a). In addition, the beneficial effect of Cu incorporation was identified by DFT calculations. According to Figure 15b,c, appropriate Cu incorporation gave rise to the ameliorated CH_3_OH adsorption and formic acid desorption abilities, which are essential for superior MOR activity. Very lately, the same group proposed and validated the pivotal role of hydroxyl vacancies in NiCo‐LDHs nanosheets, which could also promote the adsorption of methanol.^[^ ^172^ ^]^ Thus, the monolayered NiCo‐LDHs nanosheets with abundant hydroxyl vacancies achieved the desired potential of 1.35/1.40 V versus RHE at 50/100 mA cm^−2^.

Except for increasing the utilization of active sites, hybridization of TM‐LDHs nanosheets with highly conductive materials is another mainstream research field for elevating MOR and EOR activities. For example, Li et al. reported a bifunctional hierarchical heterostructured Co*_x_* P@NiCo‐LDHs for efficient water/methanol electrolysis.^[^ ^173^ ^]^ Owing to rapid electron transfer and highly exposed active sites, the current density toward MOR was obviously higher than that of counterpart materials (Figure 15d). Also, the heterostructure resulted in extremely high FEs (≈100%) at various current densities (Figure 15e). Furthermore, Co*_x_* P@NiCo‐LDHs could be used as anodic and cathodic materials simultaneously in a symmetrical electrolyzer. As seen in Figure 15f, the voltage for hydrogen generation was reduced by 242 mV with the existence of methanol in alkaline electrolyte. Yang et al. constructed various NiFe‐LDHs/carbon nanotube (CNT) composites with a classical hydrothermal method for efficient EOR.^[^ ^174^ ^]^ Figure 15g clearly shows that NiFe‐LDHs nanosheets were closely formed on the surface of spherical NiFe‐LDHs/CNT composite. In comparison of CV and chronoamperometric analysis results (Figure 15h,i), the relatively high current density of NiFe‐LDHs/CNT composite was originated from the positive effect of CNTs, which could accelerate charge transfer and provide robust mechanical strength. Albeit TM‐LDHs nanosheets have been investigated to some extent as MOR/EOR electrocatalysts (**Table** **5**), most existing researches related to TM‐LDHs nanosheets remain at the stage of material design. Similar to UOR, the RDS of product desorption limits the whole kinetics of MOR/EOR. Thus, a comprehensive understanding of MOR/EOR mechanism on the surface of TM‐LDHs nanosheets should be deciphered to design novel TM‐LDHs nanosheets‐based electrocatalysts.

**Table 5:** Recent reported TM‐LDHs nanosheets for MOR/EOR

Materials	Methanol/ethanol concentration	Potential [V vs RHE]	Current density [mA cm^−2^]	Refs.
Ni_0.33_Co_0.67_(OH)_2_	0.5 m Methanol	1.33	10	[170]
Cu_0.33_CoCo‐LDHs	3 m Methanol	1.34	50	[171]
NiCo‐LDHs	3 m Methanol	1.35	50	[172]
Co*_x_* P@NiCo‐LDHs	0.5 m Methanol	1.32	50	[173]
CeO_2_/NiV–LDHs	0.5 m Methanol	1.61	240	[175]
NiFe‐LDHs@SnO_2_	0.5 m Methanol	1.40	10	[176]
NiFe*_x_* P@NiCo‐LDHs	0.5 m Methanol	1.40	50	[177]
NiAl‐LDHs	1 m Ethanol	1.59	45.8	[18c]
NiFe‐LDHs/CNT	1 m Ethanol	1.56	30.5	[174]

## 6 Biomass Derivatives Oxidation

Biomass is regarded as an ideal substitute for fossil resources to manufacture commercial industrial products.^[^ ^178^ ^]^ Compared with traditional thermocatalytic technique, electrocatalysis possesses unique merits including benign operation condition, high efficiency, environmental friendliness, and the absence of additional reaction agents.^[^ ^179^ ^]^ Motivated by these distinct advantages, tremendous research attention has been concentrated on electrocatalytic conversion of biomass derivatives to high‐value‐added products.^[^ ^180^ ^]^ In this section, the application of TM‐LDHs nanosheets for the electro‐oxidation of specific biomass derivatives including 5‐hydroxymethylfurfural (HMF), furfural (Fur)/furfural alcohol (FFA), glycerol, and benzyl alcohol are explicitly discussed.

### 6.1 5‐Hydroxymethylfurfural

In the last few decades, HMF has attracted great interest as one of top ten biobased chemicals by the US Department of Energy.^[^ ^181^ ^]^ Since an aldehyde group and a hydroxymethyl group coexist in one HMF molecule, HMF can be further transformed into a number of valuable products, bridging the gap between the biomass resources and the chemical industry.^[^ ^182^ ^]^ Among these important products, 2,5‐furandicarboxylic acid (FDCA) have drawn extensive attention due to its usage in the production of poly(ethylene 2,5‐furandicarboxylate), a prospective substitute for petroleum‐based polyethylene terephthalate.^[^ ^183^ ^]^

**Figure 16:**
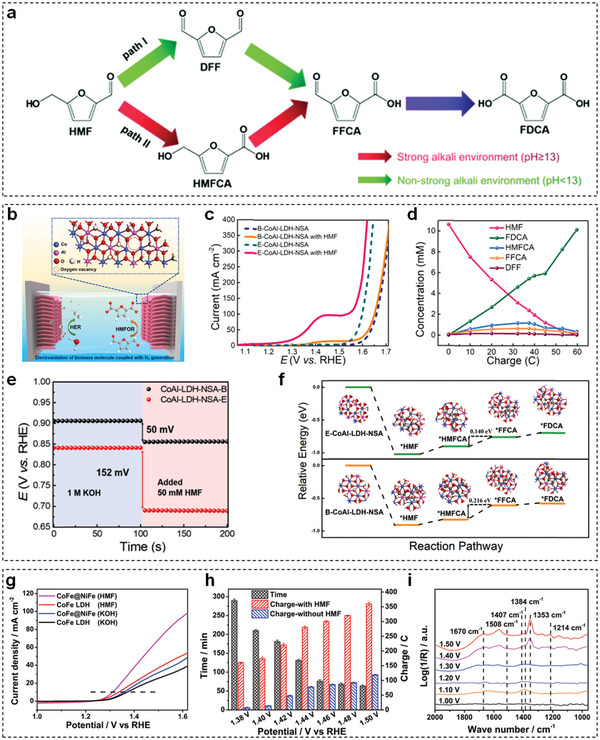
a) Two pathways of electrochemical oxidation of HMF. Reproduced with permission.^[^ ^184a^ ^]^ Copyright 2021, Royal Society of Chemistry. b) Illustration of E‐CoAl‐LDH‐NSA for electro‐oxidation of HMF coupled with hydrogen generation. c) LSV curves of B‐CoAl‐LDH‐NSA and E‐CoAl‐LDH‐NSA in 1 m KOH with and without 10 × 10^−3^ m HMF. d) Concentration of HMF and corresponding oxidation products with increasing charge for E‐CoAl‐LDH‐NSA. e) OCP comparison of B‐CoAl‐LDHs‐NSA and E‐CoAl‐LDHs‐NSA with the addition of 50 × 10^−3^ m HMF after 100 s. f) Free energy diagrams of HMF electro‐oxidation to FDCA on E‐CoAl‐LDH‐NSA and B‐CoAl‐LDH‐NSA. Reproduced with permission.^[^ ^186^ ^]^ Copyright 2021, Elsevier. g) LSV curves of CoFe@NiFe and CoFe‐LDHs nanosheets in 1 m KOH with and without 10 × 10^−3^ m HMF. h) Comparison of the total time for HMF conversion, and the consumed charge with and without HMF at different potentials. i) In situ Raman spectra of CoFe@NiFe electrocatalyst at different potentials during HMF electro‐oxidation. Reproduced with permission.^[^ ^188^ ^]^ Copyright 2021, Wiley.

Generally, HMF is oxidized to FDCA through two possible pathways, which are illustrated in **Figure** **16**a.^[^ ^184^ ^]^ The first step of path I and path II is the oxidation of the hydroxymethyl group and the aldehyde group of HMF to form 2,5‐diformylfuran (DFF) and 5‐hydroxymethyl‐2‐furancarboxylic acid (HMFCA) intermediates, respectively. Then both DFF and HMFCA intermediates in these two pathways are further oxidized to formyl‐2‐furancarboxylic acid (FFCA) intermediate and final product of FDCA. Depending on the adsorption ability of the hydroxymethyl group and the aldehyde group of HMF, HMF electro‐oxidation process is highly dependent on pH value. Specifically, in a strong alkaline environment (pH ≥ 13), the aldehyde group of HMF is more prone to be absorbed on the electrocatalyst surface in comparison of the hydroxymethyl group, resulting in HMFCA as the dominant intermediate. Conversely, in neutral or acidic environment (pH < 13), the hydration of the aldehyde group is inhibited, denoting that path I is a more favorable pathway.

TM‐LDHs nanosheets have been gradually deemed as efficient electrocatalysts for HMF electro‐oxidation. Liu et al. first used NiFe‐LDHs nanosheets to produce FDCA through electrochemical oxidation of HMF with both high yield (98%) and FE (99.4%).^[^ ^185^ ^]^ Afterward, Song et al. employed an electrochemical exfoliation method to construct oxygen‐deficient CoAl‐LDHs nanosheet array (E‐CoAl‐LDH‐NSA) for efficient electro‐oxidation of HMF (Figure 16b).^[^ ^186^ ^]^ Taking advantages of more exposed active sites and rich oxygen vacancies, the electrocatalytic performance of E‐CoAl‐LDHs‐NSA toward HMF oxidation outperformed that of bulky CoAl‐LDHs nanosheet array (B‐CoAl‐LDHs‐NSA) (Figure 16c). According to the product analysis in Figure 16d, HMFCA and FFCA intermediates were observed during the electro‐oxidation process, indicating that path II was preferentially occurred on E‐CoAl‐LDH‐NSA. More importantly, the beneficial role of oxygen vacancy was clarified by open circuit potential (OCP) tests (Figure 16e) and free energy diagrams (Figure 16f). Compared with B‐CoAl‐LDHs‐NSA, a larger OCP difference and a smaller energy barrier of the dehydrogenation step for E‐CoAl‐LDHs‐NSA demonstrated that oxygen vacancies can enhance the adsorption of HMF and accelerate the kinetics of RDS, respectively, giving rise to high FDCA yield. In addition to binary LDHs, Zhang et al. reported ternary NiCoFe‐LDHs nanosheets as efficient electrocatalysts for HMF oxidation.^[^ ^187^ ^]^ The introduction of Fe^3+^ into NiCo‐LDHs nanosheets could reduce the interaction between metallic layers to decrease their thickness, and also modulate the electronic environment around active center. As a result, 95.5% HMF was converted to FDCA with a yield of 84.9% on NiCoFe‐LDHs nanosheets within 1 h.

Apart from pristine TM‐LDHs nanosheets, Xie et al. constructed CoFe@NiFe composites by coating NiFe‐LDHs nanosheets on CoFe‐LDHs nanosheets for HMF electro‐oxidation.^[^ ^188^ ^]^ Compared with the LSV curve of CoFe‐LDHs nanosheets (Figure 16g), the CoFe@NiFe electrocatalyst exhibited better electrocatalytic activity due to their greater surface area, higher electronic conductivity, and faster reactant transport. Afterward, within the potential range from 1.38 to 1.50 V versus RHE, the charge for HMF conversion and OER gradually increased (Figure 16h), implying that OER was competitive with HMF oxidation at a high potential range. Thus, the optimal potential of HMF oxidation was chosen as 1.40 V versus RHE and FDCA with 100% selectivity was realized at this potential with an FE of 99.8%. Furthermore, in situ Raman spectra at different potentials (Figure 16i) revealed that Ni^3+^ was the main active species during HMF electro‐oxidation.

### 6.2 Furfural/Furfuryl Alcohol

**Figure 17:**
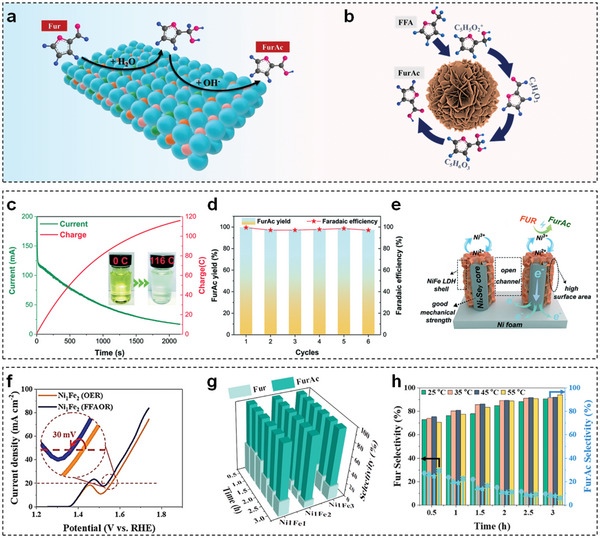
The pathway of electro‐oxidation of a) Fur to FurAc. Reproduced with permission.^[^ ^192^ ^]^ Copyright 2021, Royal Society of Chemistry. b) FFA to FurAc. Reproduced with permission.^[^ ^194^ ^]^ Copyright 2021, KeAi Publishing Communications Ltd. c) Current–time curve of Fur oxidation on the Ni*_x_* Se*_y_*‐NiFe‐LDHs electrocatalyst under controlled potential. d) FurAc yield and FE of the Ni*_x_* Se*_y_*‐NiFe‐LDHs electrocatalyst after six cycles. e) The mechanism proposed for Fur electro‐oxidation on the Ni*_x_* Se*_y_*‐NiFe‐LDHs electrocatalyst. Reproduced with permission.^[^ ^195^ ^]^ Copyright 2022, Royal Society of Chemistry. f) LSV curves of NiFe‐LDHs nanosheets in 1 m KOH with and without FFA. The selectivity of products on NiFe‐LDHs nanosheets with g) different Ni^2+^/Fe^3+^ ratio and h) different reaction temperature. Reproduced with permission.^[^ ^194^ ^]^ Copyright 2021, KeAi Publishing Communications Ltd.

Fur is a lucrative hemicellulose‐derived substance for various applications, such as pharmaceuticals, oil refining, and pesticides.^[^ ^189^ ^]^ The traditional route for Fur oxidation is proceeded at both high‐temperature and high‐pressure oxygen using oxidants or noble metal catalysts (e.g., Pt, Au, Pd, Ru).^[^ ^190^ ^]^ Alternatively, Fur electrochemical oxidation is an attractive replacement to the conventional thermocatalytic technique since the new oxidation process can be operated in normal pressure and temperature without adding external oxygen.^[^ ^191^ ^]^ As seen in **Figure** **17**a, Fur could be electro‐oxidized to furoic acid (FurAc) by converting the aldehyde group to carboxylic acid.^[^ ^192^ ^]^ However, Cannizzaro reaction is always proceeded in Fur electro‐oxidation process under alkaline condition, leading to an undesired FurAc yield (<50%).^[^ ^193^ ^]^ Therefore, further oxidation of another product in Cannizzaro reaction, FFA, becomes important for increasing FurAc yield. Figure 17b clearly depicts the pathway of FFA oxidation.^[^ ^194^ ^]^

Very recently, TM‐LDHs nanosheets are reported as the efficient electrocatalysts for FurAc generation. For example, Fan et al. assembled NiFe‐LDHs nanosheets on Ni*_x_* Se*_y_* nanowire arrays to construct core–shell Ni*_x_* Se*_y_*‐NiFe‐LDHs electrocatalyst for Fur electro‐oxidation.^[^ ^195^ ^]^ As displayed in Figure 17c, the conversion of Fur was complete after passing 116 C to obtain FurAc yield of 99.7% and FE of 99.5% on this core–shell electrocatalyst. Afterward, the FurAc yield and FE exhibited inconspicuous degradation after six cycles, demonstrating robust stability (Figure 17d). Herein, the satisfied electrocatalytic performance for FF electro‐oxidation was attributed to the following advantages (Figure 17e): the highly conductive Ni*_x_* Se*_y_* nanowires facilitated electron transport and provided robust mechanical support; NiFe‐LDHs nanosheets with a large surface area offered sufficient active sites; and the open structure ensured electrolyte penetration. Liu et al. synthesized 3D hierarchical NiFe microflowers as the efficient electrocatalyst via electrodeposition strategy.^[^ ^194^ ^]^ As depicted in Figure 17f, the selective oxidation of FFA occurred in advance of OER. By adjusting preparation and testing parameters, optimal NiFe‐LDHs nanosheets achieved the highest FFA conversion of 81.4% and FurAc selectivity of 94.0% within 3 h (Figure 17g,h).

### 6.3 Glycerol

**Figure 18:**
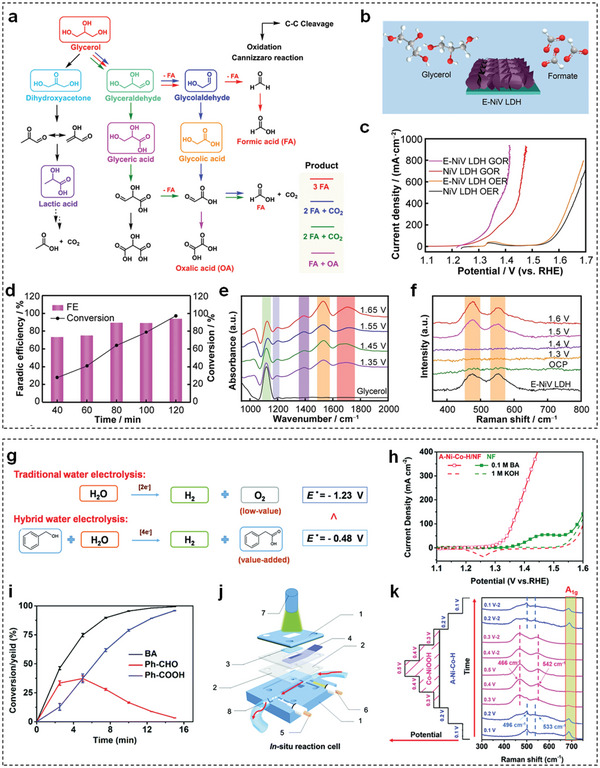
a) Reaction pathways for the electro‐oxidation of glycerol. Reproduced with permission.^[^ ^199^ ^]^ Copyright 2022, Wiley. b) Scheme of E‐NiV‐LDHs nanosheets for production of formate. c) LSV curves of E‐NiV‐LDHs and NiV‐LDHs nanosheets in 1 m KOH with and without 0.1 m glycerol. d) Time‐dependent glycerol conversion and FE on E‐NiV‐LDHs nanosheets at 1.46 V versus RHE. e) In situ FTIR spectra at different potentials during GOR. f) In situ Raman spectra of E‐NiV‐LDHs nanosheets at different potentials during GOR. Reproduced with permission.^[^ ^201^ ^]^ Copyright 2022, Nonferrous Metals Society of China. g) Reaction pathways for the electro‐oxidation of benzyl alcohol. h) LSV curves of NiCo‐LDHs nanosheets in 1 m KOH with and without 0.1 m benzyl alcohol at 10 mV s^−1^. i) Time‐dependent conversion/yield in benzyl alcohol electro‐oxidation reaction at a potential of 1.5 V versus RHE. j) The schematic illustration of the in situ reaction cell. k) In situ Raman spectra of NiCo‐LDHs nanosheets under different potentials. Reproduced with permission.^[^ ^206^ ^]^ Copyright 2020, Royal Society of Chemistry.

Glycerol, a C3 platform molecule, is the main by‐product from biodiesel production.^[^ ^196^ ^]^ Numerous high‐value‐added products could be obtained by selective oxidation of glycerol, including dihydroxyacetone, glyceric acid, tartronic acid, mesoxalic acid, glycolic acid, and formic acid.^[^ ^197^ ^]^ Unlike traditional thermocatalytic glycerol oxidation reactions (GOR), electrocatalytic GOR is a promising route, which can proceed under moderate temperature and atmospheric pressure with low cost and high efficiency.^[^ ^198^ ^]^ Electrocatalytic GOR is a multielectron transfer process, which is generally classified into two reaction routes. As described in **Figure** **18**a, the rightward direction and downward direction correspond to the cleavage of C—C bond and oxidation of alcohol to aldehyde or carboxylic acid through PCET, respectively. 1) Oxidation of alcohol into aldehyde or carboxylic acid through PCET (downward direction).^[^ ^199^ ^]^ For formic acid production, the theoretical FE of one pathway with glyceraldehyde and glycolaldehyde intermediates is achieved as 100%, whereas maximum FE of other pathways is 66.6%.

Noble metals are most common electrocatalysts for GOR.^[^ ^200^ ^]^ Lately, TM‐LDHs nanosheets have been utilized as low‐cost replacements to these precious electrocatalysts for GOR. For example, Dong et al. developed an electrochemical treatment on NiV‐LDHs nanosheets to generate plenty of Ni(III) active sites for efficient GOR (Figure 18b).^[^ ^201^ ^]^ Electrochemically regulated NiV‐LDHs (E‐NiV‐LDHs) nanosheets presented superior GOR activity with a low potential of 1.23 V versus RHE to drive 10 mA cm^−2^ (Figure 18c). After optimizing the potential for GOR, high glycerol conversion of 98% and FE of 94% were demonstrated (Figure 18d). Subsequently, GOR mechanism of E‐NiV‐LDHs nanosheets was further investigated by in situ techniques. In situ Fourier transform infrared (FTIR) and Raman spectra in Figure 18e,f revealed that the final product formate should be formed during GOR process and Ni(III) active sites were responsible for high‐efficient formate generation, respectively. Similarly, NiCo‐LDHs nanosheets were also validated as the efficient GOR electrocatalysts.^[^ ^202^ ^]^ Intriguingly, it is found that the formation of oxygen vacancies on the surface of NiCo‐LDHs nanosheets during GOR was conducive to the cleavage of the 2nd C—C bond. Therefore, NiCo‐LDHs nanosheets displayed excellent GOR activity to afford 100 mA cm^−2^ at 1.35 V versus RHE with formate selectivity of 94.3%.

### 6.4 Benzyl Alcohol

Benzaldehyde and benzoic acid are important high‐value‐added chemicals that are extensively employed in pharmaceutical, antiseptic, and agrochemical industries.^[^ ^203^ ^]^ Conventional industrial synthesis of benzaldehyde and benzoic acid from benzyl alcohol always involves the utilization of toxic reagents under harsh condition.^[^ ^204^ ^]^ Therefore, it is extremely desired to develop a new sustainable catalytic oxidation method for benzyl alcohol. Benzyl alcohol electro‐oxidation is an emerging technique with the benefits of benign operation conditions and environmental friendliness.^[^ ^205^ ^]^ A classical pathway of benzyl alcohol electro‐oxidation is shown in Figure 18g.^[^ ^206^ ^]^ The theoretical potential of benzyl alcohol electro‐oxidation and OER are −0.48 and −1.23 V, respectively, revealing that benzyl alcohol electro‐oxidation reaction is more advantageous over OER in the thermodynamic aspect. Nevertheless, most electrocatalysts for benzyl alcohol electro‐oxidation are suffered from insufficient current density, which cannot meet the industrial requirement.^[^ ^207^ ^]^

Recently, amorphous NiCo‐LDHs nanosheets were used as brilliant high‐performance electrocatalyst for the electro‐oxidation of benzyl alcohol to benzoic acid.^[^ ^206^ ^]^ As displayed in Figure 18h, the as‐prepared NiCo‐LDHs nanosheets realized an ultra‐large current density over 400 mA cm^−2^ before the appearance of OER. At the maximum potential of 1.5 V versus RHE, 99% benzyl alcohol was converted to benzoic acid with a high yield of ≈99% within 15 min (Figure 18i). Furthermore, in situ Raman measurements were carried out on NiCo‐LDHs nanosheets to decipher the actual active center during electro‐oxidation process. The corresponding reaction cell was designed as Figure 18j. As the anodic potential was cycled between 0.2 and 0.5 V versus Ag/AgCl, the occurrence of characteristic peaks at 466 and 542 cm^−1^ confirmed that NiOOH was the active species in the benzyl alcohol oxidation reaction (Figure 18k). This work sheds the light on designing the electrocatalysts for biomass‐derived oxidation with industrial level.

Summarily, TM‐LDHs nanosheets can be regarded as one of the most active materials for biomass derivatives oxidation. **Table** **6** lists recent reported TM‐LDHs nanosheets as efficient electrocatalysts for various biomass derivatives oxidation. However, several problems still need to be resolved. The first problem is to uncover the active center of TM‐LDHs nanosheets more distinctly. The second problem is the limited stability of TM‐LDHs nanosheets with the prolonged exposure to strong alkaline electrolyte. Hence, in order to further enhance electrocatalytic performance of TM‐LDHs nanosheets for biomass derivatives oxidation, new strategies should be developed in both experimental and theoretical aspects. Furthermore, since *OH intermediate is involved in electrocatalytic oxidation of biomass derivatives,^[^ ^11^, ^208^ ^]^ the binding energy between *OH and TM‐LDHs nanosheets should be optimized in the future material design.

**Table 6:** Recent reported TM‐LDHs nanosheets for biomass oxidations

Materials	Electrolyte	Yield [%]	FE [%] @ E [V vs RHE]	Ref.
NiFe‐LDHs	1 m KOH/10 × 10^−3^ m HMF	FDCA, 98.0	99.4 @ 1.23 V	[185]
CoAl‐LDHs	1 m KOH/10 × 10^−3^ m HMF	FDCA,/	99.4 @ 1.52 V	[186]
d‐NiFe‐LDHs	1 m KOH/10 × 10^−3^ m HMF	FDCA, 96.8	84.47 @ 1.48 V	[209]
NiCoFe‐LDHs	1 m NaOH/10 × 10^−3^ m HMF	FDCA, 84.9	≈90 @ 1.52 V	[187]
NiCoMn‐LDHs	1 m NaOH/1 × 10^−3^ m HMF	FDCA, 91.7	≈65 @ 1.50 V	[192]
Cu*_x_* S@NiCo‐LDHs	1 m KOH/10 × 10^−3^ m HMF	FDCA, ≈99.0	99.0 @ 1.32 V	[210]
CoFe@NiFe	1 m KOH/10 × 10^−3^ m HMF	FDCA, 99.8	99.8 @ 1.40 V	[188]
NiCoMn‐LDHs	1 m NaOH/1 × 10^−3^ m Fur	FurAc, 92.4	≈70 @ 1.50 V	[192]
Ni*_x_* Se*_y_*‐NiFe LDHs	1 m KOH/20 × 10^−3^ m Fur	FurAc, 99.7	99.5 @ 1.423 V	[195]
NiFe‐LDHs	1 m KOH/1 × 10^−3^ m FFA	FurAc,/	90.4 @ 1.60 V	[194]
E‐NiV‐LDHs	1 m KOH/0.1 m glycerol	Formate, 94.0	94 @ 1.46 V	[201]
NiCo‐LDHs	1 m KOH/0.1 m glycerol	Formate, 94.3	≈100 @ 1.474 V	[202]
A‐NiCo‐LDHs	1 m KOH/0.1 m benzyl alcohol	Benzyl alcohol, 99.0	93.5 @ 1.50 V	[206]

## 7 Summary and Perspective

In this review, we have discussed the synthetic strategies in preparing TM‐LDHs nanosheets, and summarized the recent advances of TM‐LDHs nanosheets for various electrocatalytic applications including OER, HER, UOR, NRR, small molecule oxidation, and biomass derivatives oxidation in details. Among these electrocatalytic applications, the mechanism and performance optimization of OER, HER, and UOR using TM‐LDHs nanosheets have been thoroughly studied. Although TM‐LDHs nanosheets have drawn tremendous attention owing to their low cost, easy accessibility, and changeable intrinsic electronic structure, there is still more space for performance improvements. Hence, the following perspectives are proposed:

1) Scale‐up: Developing cost‐effective fabrication methods of large‐scale TM‐LDHs nanosheets‐based electrodes should be a prerequisite for advancing toward industrialization. Nevertheless, most current fabrication processes are restricted to the lab‐scale level. Few researchers have conducted fundamental studies on the architecture of electrodes with industrial‐level size. Hence, the design criteria of TM‐LDHs nanosheets from the lab to industry should be better understood.
2) Substrate: Due to high surface energy, aggregation of TM‐LDHs nanosheets unavoidably occurs without using any substrate, leading to a slow electron transfer, limited contract between the electrolyte and electrocatalyst, and suppressed electrocatalytic activity of active sites. There is an emerging trend to prepare self‐supporting electrocatalysts by in situ growing TM‐LDHs nanosheets on highly conductive 3D substrates to improve conductivity and stability. Nowadays, the main types of substrates are NF, cobalt foam, and carbon paper. For maximizing the excellent performance of TM‐LDHs nanosheets, it is also essential to build a substrate with a quick desorption rate for products.
3) Surface reconstruction: Intensive surface reconstruction has been identified on TM‐LDHs nanosheets during electrocatalytic process, resulting in true active center for the enhanced electrocatalytic activity. However, the fundamental origin of the self‐reconstruction of TM‐LDHs nanosheets and the factors governing the relationship between the self‐reconstruction and electrocatalytic performance are not fully unveiled. Thus, to better design TM‐LDHs nanosheets‐based electrocatalysts, in situ/operando characterization techniques (e.g., electrochemistry or microscopy/spectroscopy) are required to uncover the mechanism of surface reconstruction at the molecular level.
4) Long‐term stability in practical ambient environments: Currently, most electrocatalytic research using TM‐LDHs nanosheets is proceeded in alkaline electrolyte with high purity. However, strong alkaline condition causes severe corrosion of electrodes and other corresponding components for a long‐term run. Also, various impurities, such as dust, and corrosive ions in the actual water could deactivate active sites and further corrode the TM‐LDHs nanosheets. Thus, it is highly desired to elucidate the electrocatalytic mechanism of TM‐LDHs nanosheets‐based electrocatalysts in neutral electrolyte solution and modify the surface of TM‐LDHs nanosheets to mitigate the corrosion in actual water.

With this comprehensive overview, we believe that the readers could follow the current trends in designing and evaluating TM‐LDHs nanosheets for electrocatalytic applications. We hope this review can provide inspiration to promote the development and application of related high‐performance electrocatalysts.

## Conflict of Interest

The authors declare no conflict of interest.
